# Strain level and comprehensive microbiome analysis in inflammatory bowel disease *via* multi-technology meta-analysis identifies key bacterial influencers of disease

**DOI:** 10.3389/fmicb.2022.961020

**Published:** 2022-10-14

**Authors:** Jayamary Divya Ravichandar, Erica Rutherford, Cheryl-Emiliane T. Chow, Andrew Han, Mitsuko Lynn Yamamoto, Nicole Narayan, Gilaad G. Kaplan, Paul L. Beck, Marcus J. Claesson, Karim Dabbagh, Shoko Iwai, Todd Z. DeSantis

**Affiliations:** ^1^Second Genome Inc., Brisbane, CA, United States; ^2^Department of Medicine, University of Calgary, Calgary, AB, Canada; ^3^School of Microbiology, University College Cork, Cork, Ireland

**Keywords:** meta-analysis, gut microbiome, inflammatory bowel disease, metagenomics, 16S rRNA, strain

## Abstract

**Objective:**

Inflammatory bowel disease (IBD) is a heterogenous disease in which the microbiome has been shown to play an important role. However, the precise homeostatic or pathological functions played by bacteria remain unclear. Most published studies report taxa-disease associations based on single-technology analysis of a single cohort, potentially biasing results to one clinical protocol, cohort, and molecular analysis technology. To begin to address this key question, precise identification of the bacteria implicated in IBD across cohorts is necessary.

**Methods:**

We sought to take advantage of the numerous and diverse studies characterizing the microbiome in IBD to develop a multi-technology meta-analysis (MTMA) as a platform for aggregation of independently generated datasets, irrespective of DNA-profiling technique, in order to uncover the consistent microbial modulators of disease. We report the largest strain-level survey of IBD, integrating microbiome profiles from 3,407 samples from 21 datasets spanning 15 cohorts, three of which are presented for the first time in the current study, characterized using three DNA-profiling technologies, mapping all nucleotide data against known, culturable strain reference data.

**Results:**

We identify several novel IBD associations with culturable strains that have so far remained elusive, including two genome-sequenced but uncharacterized *Lachnospiraceae* strains consistently decreased in both the gut luminal and mucosal contents of patients with IBD, and demonstrate that these strains are correlated with inflammation-related pathways that are known mechanisms targeted for treatment. Furthermore, comparative MTMA at the species *versus* strain level reveals that not all significant strain associations resulted in a corresponding species-level significance and conversely significant species associations are not always re-captured at the strain level.

**Conclusion:**

We propose MTMA for uncovering experimentally testable strain-disease associations that, as demonstrated here, are beneficial in discovering mechanisms underpinning microbiome impact on disease or novel targets for therapeutic interventions.

## Introduction

Most microbiome studies in human diseases are based upon single-cohort, single-technology analyses of microbial populations to produce associations between bacterial groups and disease with limited strain identification. The results can potentially be biased by disparities in clinical protocols, cohort demographics, molecular analysis technologies, and taxonomic resolution (Walters et al., 2014; Gilbert et al., 2018). Recent meta-analyses have identified taxa, typically limited to the genus-level (Wirbel et al., 2021) that correlate with a given disease across cohorts and are often confined to single technology-based analyses (Walters et al., 2014; Duvallet et al., 2017). Appreciation of strain-specific markers in single-technology meta-analysis workflows has been noted (Pasolli et al., 2016), and given that biological activity can be strain-specific, knowledge of specific strains is fundamental to deciphering their functional role in disease (De Filippis et al., 2019). For example, a metagenomic survey of stool from patients with IBD identified a bloom of specific *Ruminococcus gnavus* strains in patients but not in control subjects, and these strains were found to harbor genes that conferred them an adaptive advantage in disease (Hall et al., 2017). There is thus a need for a systematic approach to identifying strains associated with health or disease consistently across the growing number of published studies. Furthermore, microbial-abundance changes inferred from single-technology meta-analyses are subject to caveats associated with each DNA-profiling technology, such as the limited taxonomic resolution from sequencing the 16S rRNA gene (16S.NGS) or sporadic detection of low-abundance taxa in whole genome shotgun sequencing (WGS.NGS) (Hamady and Knight, 2009; Shah et al., 2011) related to limited sampling depth. These limitations have restricted our view of the microbiome’s role in disease to higher-order taxa and to those inferred from datasets characterized by a single technology. In this study, we develop and propose multi-technology meta-analysis (MTMA) as a platform for aggregation of independently generated datasets from multiple DNA-profiling technologies to facilitate a comprehensive strain-level view of disease and apply it to the large number of microbiome datasets available in IBD.

Patients with IBD suffer from chronic inflammation of the gastrointestinal tract where genetic, environmental, immune, and microbial factors have all been implicated in disease onset and progression. Recent metagenomic surveys of the microbiome in IBD (Vich Vila et al., 2018; Franzosa et al., 2019; Lloyd-Price et al., 2019) at the species and strain-level present an important advancement in understanding the functional role of the microbiome in IBD and present a rich source of data that can be systematically analyzed to identify cross-cohort strain-IBD associations. We present the first strain-level analysis of IBD, where we have mapped all data against DNA records for known, isolated, and named strains, integrating microbiome datasets characterized using three DNA-profiling technologies and pinpoint strains enriched or reduced in disease by applying a novel MTMA technique on 21 datasets. We identified previously unreported associations that were either unique to IBD subtypes [Ulcerative Colitis (UC) and Crohn’s Disease (CD)] or persisted across subtypes and even gut ecosystems (microbiome associated with stool/luminal-contents and intestinal-mucosa/biopsy samples from the large-intestine). Herein, we discovered *Alistipes putredinis*, a *Gemminger formicilis* and two as yet uncharacterized *Lachnospiraceae* strains that were decreased in both the lumen and mucosal contents of patients with UC and CD across multiple cohorts. Identification of the ubiquitous decrease of these strains in IBD supports the notion that these bacteria likely play critical roles in the healthy human guts and presents opportunities for the development of IBD diagnostic or therapeutic interventions that aim to restore these strains or their associated functions to levels in healthy subjects. Furthermore, we confirmed previously observed associations in IBD and reported the applicability of these findings across geographically dispersed cohorts. The work presented herein is the largest and most comprehensive integration of microbiome in IBD to our knowledge and demonstrates the ability of MTMA to build upon previous work in this field and uncover strain-disease associations that can be further elucidated for their functional role in IBD.

## Methods

The cohorts integrated to demonstrate the MTMA methodology was required to contain both control subjects and UC or CD cases or both. Each patient was linked to either a stool biospecimen or a mucosal biopsy biospecimen or both. Supplementary Table 1 lists the laboratory techniques used to profile the biospecimens, the counts of cases and controls, and the depth of sequencing.

## Patient populations and sample collections for SG-Cohort 2_2013

Mucosal biopsies were collected at the University of Calgary during initial diagnosis or follow-up endoscopy from September 2007 to April 2013 from control and UC subjects (BioProject accession number PRJNA527097). A diagnosis of UC was confirmed by a qualified gastroenterologist at the Foothills Medical Center, University of Calgary. Informed consent for use of biopsy samples was obtained by the University of Calgary Intestinal Inflammation Tissue Bank and the study was approved by the University of Calgary, Conjoint Health Research Ethics Board (ID: 18142 and 14-2429).

## Generation of microbiome profiling data for SG-Cohort 2_2013

### DNA isolation

DNA was isolated from mucosal biopsies using the MoBio Ultraclean Tissue and Cells DNA isolation kit (MoBio Laboratories, Carlsbad, CA, USA) following instructions provided by the manufacturer.

### 16S.NGS data generation

The 16S rRNA V4 region was PCR-amplified using fusion primers designed against surrounding conserved regions and tailed to incorporate Illumina adaptors and indexing barcodes as described previously (Caporaso et al., 2012). Amplicons were sequenced on the Illumina MiSeq (Illumina, San Diego, CA, USA) following instructions provided by the manufacturer.

### PhyloChip data generation

V1 through V9 16S rRNA gene analysis was performed on the G3 PhyloChip (Hazen et al., 2010; Mendes et al., 2011) using lab protocols and image-scoring procedures previously described (Sarhan et al., 2018).

## Generation of RNAseq data for SG-Cohort 1_2014

Biopsies were completely defrosted in RNA-later before performing RNA purification with the AllPrep RNA Mini kit (Qiagen). Defrosted biopsies were transferred into a tube containing 350 μl RLT buffer with β-mercaptoethanol (Sigma-Aldrich, St Louis, MO, USA), three 3.5 mm glass beads, and 0.25 ml of 0.1 mm glass beads (Biospec, Bartlesville, OK, USA). Disruption and homogenization were carried out in a MagNA Lyser (Roche, Penzberg, Germany) two times for 15 s at 3,500 or 6,500 rpm. RNA purification was performed according to the kit manufacturer’s instructions. DNA contaminations in RNA samples were removed by Turbo DNA-free kit following the manufacturer’s instructions (Ambion, Carlsbad, CA, USA). RNA concentrations were measured using a Nano-Drop 2000 Spectrophotometer (Thermo Scientific, Waltham, MA, USA). RNA integrity was checked on 1% agarose gel electrophoresis and 2100 Bioanalyzer system (Agilent Technologies, Santa Clara, CA, USA). In addition, RNA quality was considered acceptable if the RNA integrity number was ≥ 6 and the rRNA ratio was ≥ 1.5. Host transcriptome RNAseq was carried out by Macrogen (Seoul, South Korea) using TruSeq Stranded mRNA Sample Prep Kit (Illumina) with Illumina HiSeq 4000 2 × 150 reads following the manufacturer’s protocol.

## Procurement of raw data and metadata curation for public datasets

For the integrated meta-analysis, we obtained 13 additional cohorts where UC and/or CD subjects were profiled with WGS.NGS and/or 16S.NGS techniques against control subjects using either mucosal biopsies or stool biospecimens (Supplementary Table 1). 16S.NGS from mucosa was available for Fastq/Fasta files and metadata were procured from public repositories. Metadata stored with raw data, such as NCBI’s RunInfo table associated with the SRA Run Selector, and/or metadata published in tables in the primary text or Supplementary files of the publications, were retrieved and manually re-annotated using a controlled vocabulary of hierarchically organized terms. An in-house database was created to store all study-related data and facilitate appropriate metadata annotation of all datasets *via* manual curation. Clinical metadata was stored in this database as a series of label:value pairs attached to the biospecimen from which the data files were generated.

## StrainSelect database

StrainSelect^1^ is a reference database of archaeal and bacterial genomic identifiers organized by strain. StrainSelect assigns a consistently formatted identifier for known strains that have been isolated and have had their genome sequenced and/or their 16S rRNA gene sequenced and shared publicly.

A single strain encapsulates all the descendants of single isolation in pure culture and is usually disseminated by a succession of cultures ultimately derived from a single colony (Boone and Castenholz, 2001). The initial process of “isolation” from a living community within a biome is an unnatural selection event that captures only a point-in-time of an evolving genome that might become altered by future natural selection events (Dijkshoorn et al., 2000). Nonetheless, these isolated strains are important tools for experimental microbiology and provide points of reference to compare to future datasets so oftentimes microbiologists, after isolating and naming a single strain from clinical or environmental material, will send replicate sub-cultures to multiple biological resource centers (BRCs), such as ATCC or DSM, who then assign their own catalog numbers. DNA sequencing institutes throughout the international scientific community procure strains from various BRCs and then upload gene or genome assemblies to public databases such as the NCBI which assigns an identifier for each assembly. Since this is a decentralized international process there has been persistent confusion about what data came from which strain. A prime example can be seen in a strain isolated from a healthy Japanese male in 2011 (Morotomi et al., 2012). The research team bestowed novel genus and species level nomenclature for the isolate which they publicized as *Christensenella minuta* YIT 12065. Two independent BRCs, DSM, and JCM, also propagated sub-cultures of this strain with their own unique catalog numbers, DSM 22607 and JCM 16072. The University of California at Davis, Beijing Genome Institute, Washington University, and South China University of Technology each procured the strain from one of the BRCs then separately sequenced the extracted DNA and submitted their optimal assembly to public databases which are now downloadable under 4 different assembly identifiers: GCF_001571425, GCF_001652705, GCF_001678855, and GCF_003628755. A novice user of these public databases can misinterpret these four assemblies as four different genomes from four different strains, but they would be incorrect. In building the StrainSelect database, we sought to overcome confusion by tracing through the synonymous identifiers for sub-cultures and genomic data records and assigning a consistently formatted identifier for the strain, which in this example is StrainSelectID: t__520.

Taxonomic placement and nomenclature were adapted from GTDB (Parks et al., 2022). In cases where strains were represented by one or more 16S rRNA genes without an available genome assembly, taxonomic placement was estimated using the sintax method (Edgar, 2018). Where formal taxonomic names were not available for genera and species, numeric provisional identifiers were assigned prefixed by “PROV.” In Figure 5 and Supplementary Figures 7–10, when GTDB recognized distinct phyla placements such as Firmicutes_A, Firmicutes_B, and Firmicutes_C for ease of visualization they were grouped as Firmicutes in the figure.

**FIGURE 1:**
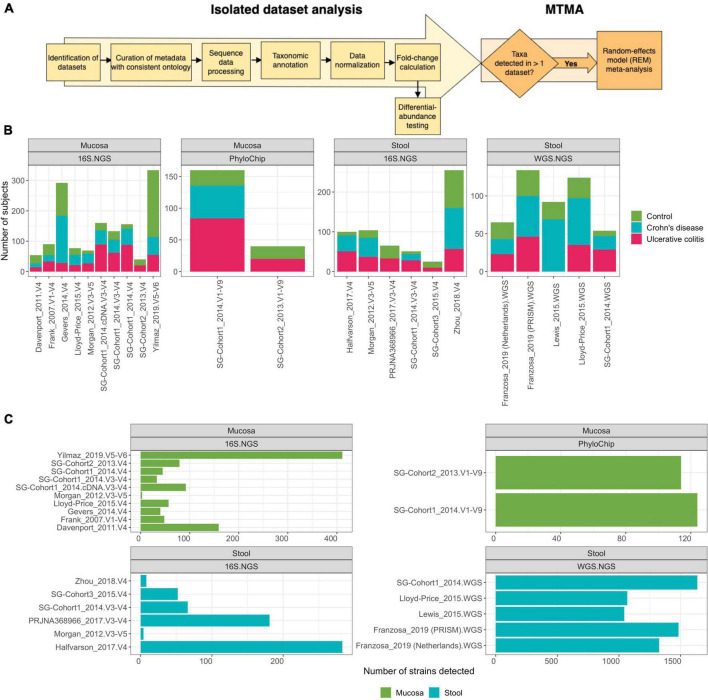
Multi-technology meta-analysis workflow and dataset characteristics. **(A)** Analysis pipeline overview. Microbiome studies in IBD with publicly available metadata and sequencing data (11 studies, until January 2020) and 3 studies generated herein were collected (Supplementary Table 1). Metadata associated with each study was manually re-annotated using a controlled vocabulary enabling comparison across datasets. Raw data from each isolated dataset was individually processed, quality-filtered, taxonomically annotated, and statistically analyzed using methods and tools appropriate for each DNA-profiling technology. Datasets were integrated using MTMA. See section “Methods” for details. **(B)** Sample size for each dataset with sub-tallies demonstrating count of control, Ulcerative colitis, and Crohn’s disease subjects. **(C)** Number of strains obtained *via* annotation against the StrainSelect database for each dataset.

**FIGURE 2:**
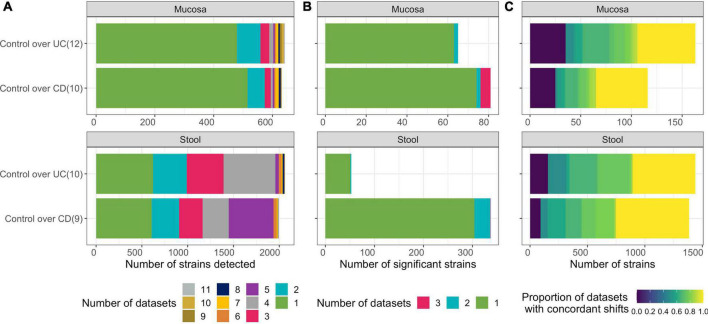
Differentially abundant strains are inconsistent across single-cohort microbiome analyses. **(A)** Number of strains detected (after prevalence filtering) in each of the two contrasts (Controls compared to UC or CD) for each microbial ecosystem (luminal/stool and mucosa) with sub-tallies colored according to the number of datasets a strain is detected in. Number of datasets considered for each contrast is specified in parentheses. **(B)** Number of strains that were significantly differentially abundant (adjusted-*p* < *0.01*) with sub-tallies colored according to the number of datasets they were significant in. **(C)** Number of strains that exhibit concordance in direction of the log-2 fold change. Color represents proportions of datasets in which they were detected within a contrast. Only strains detected in at least two isolated datasets are included.

**FIGURE 3:**
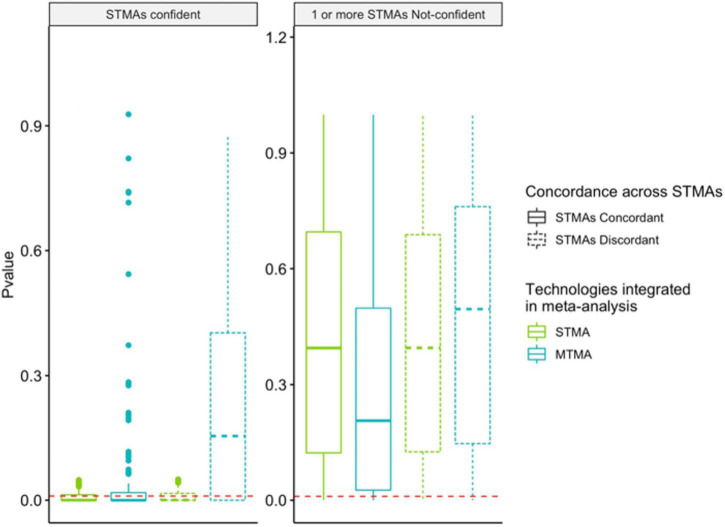
Multi-technology meta-analysis identifies significant findings that are concordant across DNA-profiling technologies, overcoming this limitation with STMAs. Permutations were run to simulate two or more observations of taxa in two or more DNA-profiling technologies. Distribution of *p*-values from Single Technology Meta-Analysis (STMA) (green; integration of simulated observations from one DNA-profiling technology) or MTMA (blue; integration of simulated observations from multiple DNA-profiling technologies) for 7,500 permuted cases is shown. Solid and dashed-boxplots correspond to cases where the direction of the log-2 fold change inferred *via* STMAs are concordant or discordant, respectively. **Left and right panels** correspond to cases where all STMAs are confident in the direction of the effect (log-2 fold change) or at least one STMA is not-confident, respectively. Confidence of an STMA effect direction was defined at 95%. Red dashed lines correspond to a *p*-value *of 0.01*.

**FIGURE 4:**
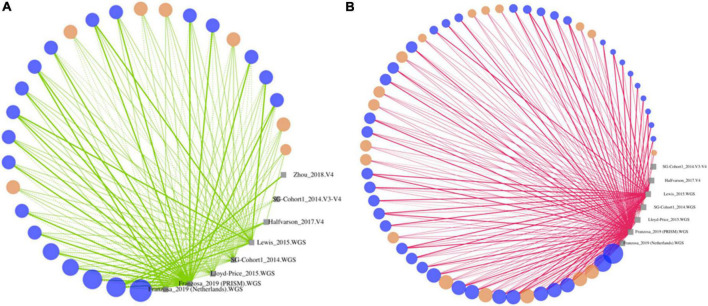
Differentially abundant strains in Crohn’s disease identified herein are concordant across cohorts. Strains that are significantly enriched **(A)** or decreased **(B)** in stool from controls compared to CD subjects based on strain-level MTMA. Strains (dots) are sized by the number of isolated datasets they are detected and colored as follows: significant by one or more isolated analyses and MTMA (blue) or MTMA only (orange). Strains are connected to isolated datasets (gray squares) they are detected in. Line color indicates enrichment (green) or decrease (pink) of strains in controls compared to case subjects. Thick and thin lines indicate significantly (adjusted *p* < *0.*01) and non-significant findings in isolated datasets, respectively. Solid and dashed lines correspond to isolated datasets that are confident or not-confident in the direction of the log-2 fold change, respectively. Confidence is determined as cases where the lower and upper bounds of the 95% confidence interval associated with a log-2 fold change are in the same direction as the log-2 fold change. Strains where the *adjusted p-value* from MTMA was < 0.0001 are shown. Supplementary Figure 2 plots strains with *adjusted p-value* in the 0.01–0.0001 range. Plots summarizing isolated dataset results for strains significant *via* MTMA for other contrasts considered herein are in Supplementary Figures 3–5.

**FIGURE 5:**
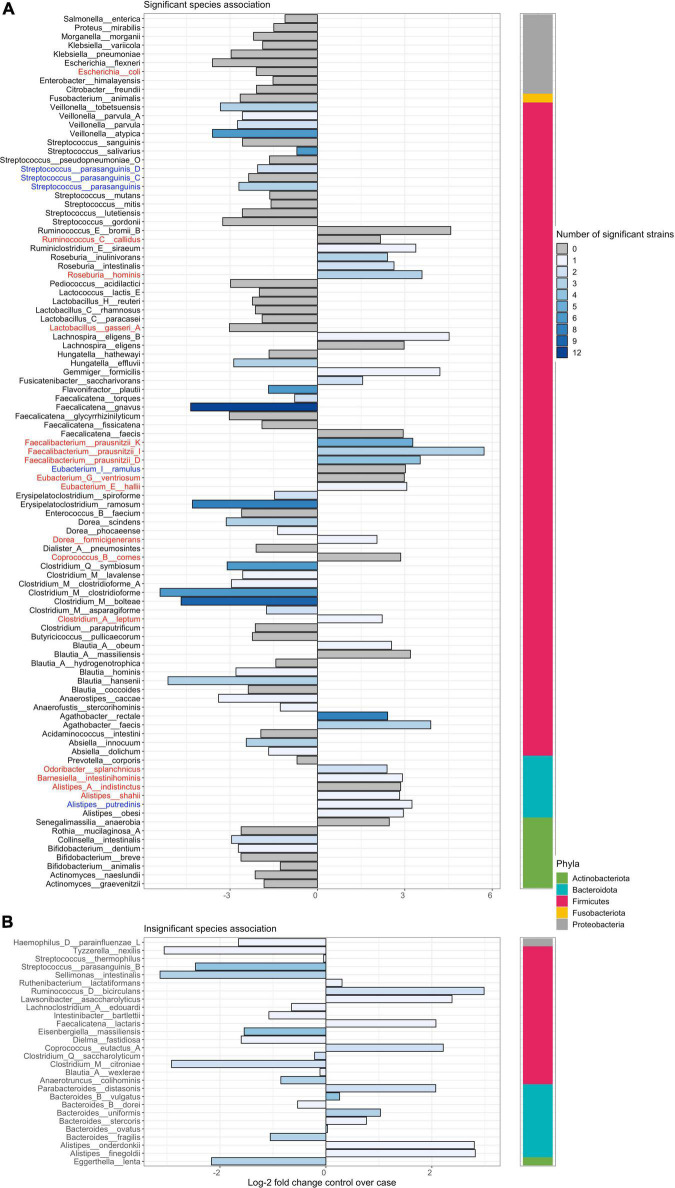
Novel dysbiosis and homeostasis-associated species and strains in the gut lumen of Crohn’s disease subjects that were previously unreported in isolated analyses. Left panel plots the log-2 fold change of differential abundance at the species level. Bars are colored by the number of strains within a species that are significantly differentially abundant (DA), with gray bars indicating cases where only species-level significance is observed. Phylum-level placement is shown in the right strip. Panel **(A)** plots cases significantly DA species with or without strain-level significance in differential abundance. Panel **(B)** plots cases with only strain-level but no species-level significant differential abundance. Significance was determined at *adjusted p* < *0.01*. Purple dots point to species that were enriched or decreased in the mucosa of CD subjects as well as stool. Species that were previously reported in the literature as DA in stool of both UC and CD patients are highlighted in blue or red. Only species that are taxonomically named are shown. Unnamed species are shown in Supplementary Figure 7. Plots summarizing results for other contrasts considered herein are provided in Supplementary Figures 8–10.

## Raw profiling data processing and taxonomic annotation

### 16S.NGS data processing

When raw sequence reads were available, they were processed *via* DADA2 applying default settings for filtering, learning errors, dereplication, amplicon sequence variant (ASV) inference, and chimera removal (Callahan et al., 2016). Truncation quality (truncQ) was set to two, and ten nucleotides were trimmed from the termini of each forward and reverse read. When only trimmed reads or only fasta files or when data was generated *via* Sanger or 454 sequencings, paired-end reads were merged (when applicable) and aligned to StrainSelect (see text footnote 1; StrainSelect19_README.txt), version 2019 (SS19) using USEARCH (Edgar, 2010) using methods described in the strain-level annotation section below. Distinct strain matches were defined as described. The remaining sequences were quality-filtered, chimera-filtered, and clustered at ≥ 97% similarity *via* UPARSE (Allali et al., 2017) to generate *de novo* OTUs. OTU abundances were generated by aligning and counting all non-strain sequences against the OTU representative sequences.

For strain-level annotation, ASVs or *de novo* OTU representative sequences were mapped to SS19 using USEARCH (Edgar, 2010) (usearch_global). SS19 is a repository of strain identifiers (and their various synonyms) and gene identifiers derived from known isolated microbial strains as of 22 July 2019. A sequence observed in a clinical biospecimen was assigned a strain-level annotation only when it met two conditions: (1) it matched at least one reference gene from one strain with ≥ 99% identity, and (2) the highest identity match to any gene from a different strain was less than that of the top strain (e.g., 99.75 vs. 99.50%). Sequence-to-strain assignment examples are provided in Supplementary Table 2. Counts of reads from all ASVs annotated uniquely to a strain were summed to obtain strain-level abundances. Similarly, in the case of the pipeline where *de novo* OTUs were generated, counts of reads from all *de novo* OTUs annotated uniquely to a strain were summed along with reads that were uniquely mapped to a strain pre-OTU generation to obtain strain-level abundances.

For species-level annotation, if a unique strain match was achieved the strain’s species level and higher level taxonomic placement was inherited. If a unique strain match was not achieved, then species level and higher taxonomic placement were estimated with sintax (-cutoff 0.80) (Callahan et al., 2016).

### PhyloChip data processing

Empirical OTUs (eOTUs) generated using image-scoring procedures were annotated against SS19 to obtain strain-level annotations as described previously (Sarhan et al., 2018; Ravilla et al., 2019).

### WGS.NGS data processing

Reads were processed with Trimmomatic (Bolger et al., 2014) to remove adapter sequences and low-quality ends (< Q20). Reads shorter than 35-bp following trimming were discarded. Contaminant sequences (e.g., sequencing primers) were removed using Bowtie (Langmead and Salzberg, 2012). Host sequences were removed *via* Kraken (Wood and Salzberg, 2014), which used exact alignments of raw shotgun sequences to k-mers derived from the human reference genome. Ribosomal RNA sequences from all three domains of life were identified and removed with SortMeRNA 2.0 (Kopylova et al., 2012). In total, short and/or low quality reads, host reads, and ribosomal RNA reads were ignored. Sourmash (Irber and Brown, 2016) was used to taxonomically annotate the remaining reads against a database built using strains in the SS19 database with genomes available as of July 2019. Strain-level annotations were summed to obtain species-level annotations.

## Statistical analyses of isolated datasets

Datasets with less than five patients remaining in either the control and UC or CD groups were excluded based on a power analysis that indicated a minimum of five subjects per group was required to detect small (log-2 fold change > 1) strain-level differential abundances when integrating five or more datasets in a meta-analysis.

### Data pre-filtering, normalization, and statistical tests

#### PhyloChip

Significant differences for all eOTUs were calculated *via* Welch’s *t*-tests, and adjusted *p*-values were determined with the Benjamini-Hochberg correction. Fold change and variance were calculated using the *metafor* package in R using the *escalc* function with measure = MD (Edgar, 2010). Standard error was calculated as the square root of the variance and both fold change and standard error were extrapolated to a log-2 scale.

#### 16S.NGS

We used methods described in ANCOM-II (Kaul et al., 2017) and used the functionality implemented in the *feature_table_pre_process* function^2^ to filter ASV or OTU tables. Briefly, this pre-filtering detected outlier values based on a cutoff of 5% (out_cut = 0.05) and performed prevalence filtering at 5% (zero_cut = 0.95). Following this a pseudo-count of 1 was added to all samples and bins, and data were normalized *via* a clr-transformation (as implemented in *clr* function in the compositions package in R). Significant differences for bins were calculated *via t*-tests, and adjusted *p*-values were determined with the Benjamini-Hochberg correction. The clr-transformation followed by the *t*-test for DA in NGS data results in high concordance across related cohorts (Wallen, 2021).

#### WGS.NGS

*f_unique_weighted* values exported per strain from sourmash were converted to the count scale. Data were then filtered and normalized using methods described for 16S.NGS. Significant differences for bins were calculated *via t*-tests, and adjusted *p*-values were determined with the Benjamini-Hochberg correction.

### Computation of effect size and standard error for meta-analysis

Whenever multiple biospecimens of the same type (stool or biopsy) were acquired from the same subject, only the earliest time point was retained. If the clinical metadata did not resolve the relative timepoints, then the biospecimen that yielded the greatest number of reads post-filtering was retained. Fold change and variance were calculated using the *metafor* package in R using the *escalc* function with measure = MD (Viechtbauer, 2010). Standard error was calculated as the square root of the variance and both fold change and standard error were extrapolated to a log-2 scale.

## Multi-technology meta-analysis

Log-2 fold change and standard errors pertaining to isolated datasets were integrated in MTMA using a Random effects model (REM) as implemented in the *rma*.mv function in the *metafor* package in R (Viechtbauer, 2010). A multi-level REM that treated cohort and dataset (combination of cohort, profiling technology, and the variable region of 16S-rRNA when applicable) as outer and inner-levels to integrate results from isolated analyses in MTMA. REM was run using the option in the rma.mv function, the Nelder-Mead optimizer with 500 maximum iterations. Only those strains or species observed in at least two datasets were retained for REM analysis. False discovery correction for REM-generated *p*-values was achieved using the Benjamini-Hochberg method.

## Permutation analysis

From a table of strain-level observations of log-2 fold changes and associated standard errors in each isolated dataset, random draws were taken to simulate a collection of observations from 1 to 3 DNA-profiling technologies, 1 to 2 biospecimen-types, 1 to 2 disease-subtype contrasts, and 2 to 21 datasets. A total of 7,500 of the random draws were made simulating 2 or 3 single-technology meta-analyses (STMAs) within an MTMA allowing comparisons between STMA and MTMA. STMA and MTMA were then run on these simulated observations as described in the MTMA section above and *p*-values, log-2 fold changes, and 95% confidence intervals were obtained for comparisons.

## Functional annotation of strains

For strains of interest, protein sequences were predicted by Prodigal (Hyatt et al., 2010). KEGG orthology (KO) annotations for the proteins were obtained using KofamKOALA (Aramaki et al., 2020). The associated pathway for these KOs was obtained from KEGG.

## Analysis of host pathways correlation with the abundance of strains of interest

### Data processing

Reads were processed with Trimmomatic (Bolger et al., 2014) to remove adapter sequences and low-quality ends (< Q20). Reads shorter than 35 bp following trimming were discarded. Contaminant sequences (e.g., sequencing primers) were removed using Bowtie (Langmead and Salzberg, 2012). HISAT2 (Kim et al., 2019) was used for mapping followed by SUBREAD (Liao et al., 2019) for feature count generation. Gene symbol conversion was performed with the Ensembl database using the *mygene* package in R (Wu et al., 2013).

### Statistical analysis for identification of enriched pathways

Mucosal biopsies from patients where both microbiome (PhyloChip) and host-expression profile were available in SG-Cohort-1, were examined for gene-expression patterns correlating with the abundance of each strain of interest (4 strains identified as significantly decreased in IBD and detected in > 75% of the contrasts examined herein; Figure 7 strains with a positive log-2 fold change by MTMA and 4 strains that were not significantly associated with IBD in this MTMA). DESeq2 (Love et al., 2014) with ashr shrinkage was used for the identification of genes that were significantly differentially abundant based on correlation to the abundance of each strain of interest. For each strain, significantly enriched pathways were determined using the pathway enrichment module in Reactome (Yu and He, 2016) as implemented in the R function *enrichPathway* from the Reactome package. Only genes that were significantly differentially abundant at an *adjusted p* < *0.05* were considered for pathway enrichment analysis. Top 20 pathways (based on *adjusted p-value*) from the pathway enrichment analysis for each strain were compared to identified pathways unique to the strains determined as significantly decreased in IBD. Note that the top 20 pathways were compared only for pathways with *adjusted p* < *0.1*.

**FIGURE 6:**
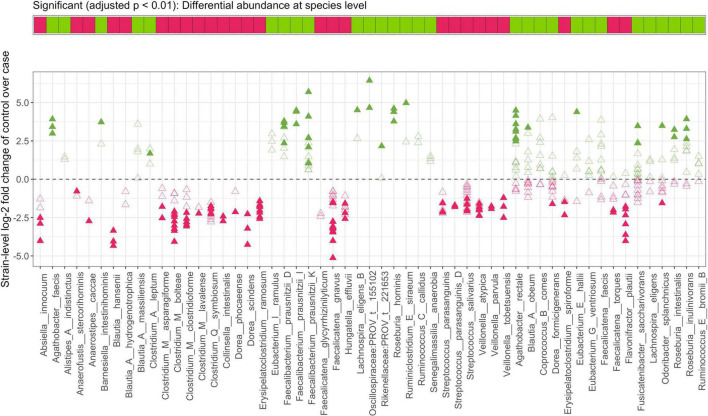
Strain-identity even within a species is important to the functional role of a bacterium in IBD. **Top panel** indicates direction of each *species*-level enrichment (green) or decrease (pink) in *stool samples from controls as compared to CD* subjects. In the **bottom panel**, log-2 fold change of strains within a species is shown with closed and open triangles indicating significant and insignificant findings in *strain*-level MTMA, respectively. Significance was determined at an *adjusted p* < *0.01*. Plots summarizing strain-level differences within a species for other contrasts considered herein are provided in Supplementary Figure 11.

**FIGURE 7:**
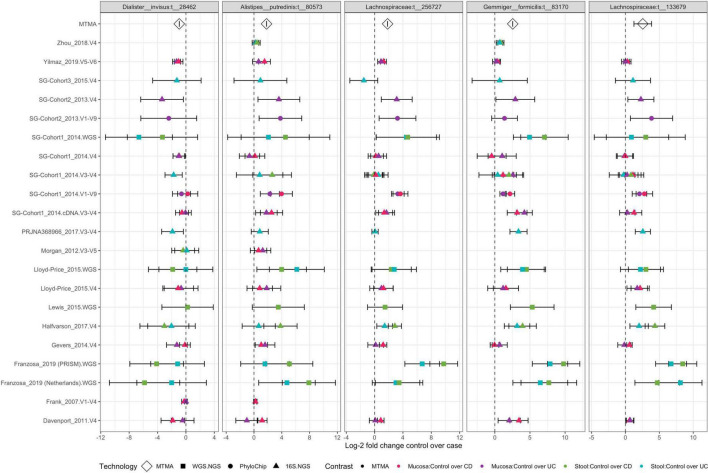
Two *Lachnospiraceae* strains are ubiquitously decreased in IBD compared to control subjects. **Each panel** plots log-2 fold change from MTMA (diamonds at the top) and isolated (squares, circles, and triangles) datasets for strains that are significantly (*adjusted p* < *0.01*) differentially abundant in an MTMA integrating all datasets and comparisons described herein. For isolated datasets, points are shaped by the DNA-profiling technology used to characterize the microbiome and colored by biospecimen type and disease subtype being compared to controls. Error bars correspond to the 95% confidence interval. Plot summarizes results for the five strains that demonstrate the most consistent association with IBD across cohorts (detected in greater than 75% of all contrasts integrated and significant by MTMA). See Supplementary Figure 15 for remaining strains identified as significant in this MTMA.

## Results

### Single cohort analyses identify study-specific microbiome signatures that are often not concordant across cohorts

To determine if associative patterns between bacterial taxa and IBD (specifically in UC and CD) in individual studies could be confirmed across cohorts, we identified microbiome studies where both clinical metadata and microbiome-sequencing data were publicly available or were generated from three of our recent studies (Supplementary Table 1). We integrated microbiome profiles that were characterized *via* WGS.NGS, 16S.NGS, or PhyloChip from 1,289 stool and 2,118 mucosa samples spanning 21 datasets across 15 cohorts using a standardized pipeline that facilitated comparison across cohorts and DNA-profiling technologies (Figures 1A,B). Patients in remission or those treated with antibiotics were excluded. Datasets with less than five patients remaining in either the control and UC or CD groups were excluded. To allow comparison of clinical variables across datasets from multiple research groups, we re-annotated metadata from public datasets using a controlled vocabulary of hierarchically organized terms. Raw data from each isolated dataset was processed, quality-filtered, taxonomically annotated, and analyzed using methods appropriate for each DNA-profiling technology. Herein we defined a strain as all the descendants of single isolation in pure culture (Boone and Castenholz, 2001). We developed StrainSelect, a curated strain database containing sequence information of bacterial and archaeal strains connected to genome identifiers (details in section “Methods”), to enable comparison of fine-scale strain variations between case and control subjects. We identified 2,626 strains across the 21 datasets with on average, 1,311 strains identified in WGS.NGS, 119 in PhyloChip, and 97 in 16S.NGS datasets (Figure 1C). These counts should not be considered as a measure of the complete taxonomic richness within the biospecimens but instead simply the counts of known strains in the reference database that were identified by stringent DNA matching. The lower number of strains was identified in 16S.NGS and PhyloChip datasets are not surprising as the ability to accurately assign 16S-rRNA sequences to a specific strain is a known limitation (Johnson et al., 2019). Thus we only identified strains that could be uniquely annotated to a sequence. When strain-level discrimination of taxa was not possible, to understand the role of these taxa in IBD, we also pursued analysis at the species level. Identification of differentially abundant (DA) strains across cohorts from a simple comparison of isolated datasets was limited as 75 or 30% of the strains were detected in only one dataset characterizing the intestinal mucosa and gut-lumen (stool), respectively (Figure 2A, green bars). Strains that were significantly DA from the isolated analyses were primarily dataset-specific (green bars, Figure 2B). Furthermore, in many cases these significantly differentially abundant strains were not always consistently associated with either homeostasis (enriched in control) or dysbiosis (enriched in case), across all datasets within a contrast (Supplementary Figure 1, rightmost panels). This sparsity in overlap of strains significantly associated with disease across datasets underscores the need for more robust means of discerning concordant signatures across cohorts. With approximately 40% of strains demonstrating concordant associations, albeit not always significant, with either homeostasis or dysbiosis (Figure 2C), we hypothesized that MTMA could enable the identification of significantly and consistently concordant taxa associations across datasets.

### Multi-technology meta-analysis identifies significant findings that are concordant across DNA-profiling technologies and is more sensitive to changes compared to STMA

We utilized a multi-level random-effects model that treated cohort and dataset as nested levels to integrate results from isolated analyses in MTMA. This enabled weighting cohorts equally even when multiple technologies were used to characterize the microbiome in a single cohort. We hypothesized that since MTMA facilitates the integration of all available microbiome data in a disease area, significant associations identified herein would represent microbes that are consistently perturbed in disease. This would eliminate findings that are not reproducible across datasets characterized by different DNA-profiling technologies which traditional single-technology meta-analysis (STMA) may fail to eliminate; given that they are restricted to trends observed in one DNA-profiling technology only. To demonstrate this added value of a comprehensive view of the microbiome in disease facilitated by MTMA compared to STMA, permutations were run to simulate two or more observations of taxa from two or more DNA-profiling technologies. For each simulated taxon, significance (*p* < *0.01*) in STMA or MTMA was determined by integrating data pertaining to each DNA-profiling technology only or all simulated data agnostic of technology (Figure 3). A taxon was considered concordant when the direction of effect (measured as log-2 fold changes) across all STMAs that evaluated the taxon were in the same direction (solid boxplots). For STMA, confidence in the reported direction of effect per taxon was determined as cases where the lower and upper bounds of the 95% confidence intervals are in the same direction as the effect. STMAs tended to identify significant associations when they were confident regardless of concordance across STMAs (left panel *p*-values are lower than the right panel in green boxplots but no significant difference in the distribution of STMA *p*-values when comparing green solid and dotted-boxplots in both panels; Figure 3). However, we observed more significant associations with MTMA when effects were concordant across DNA-profiling technologies compared to when they were discordant (greater counts of DA strains below a given *p*-value cutoff blue-solid compared to blue-dotted boxplots in both panels; Figure 3). MTMA also identified significant findings in cases where STMA were concordant even when individual STMAs were not confident in the direction of the association and failed to infer a significant STMA finding (greater counts of DA strains below a given *p*-value cutoff in MTMA compared to STMA in solid boxplots in the right panel; Figure 3). By integrating datasets across DNA-profiling technologies, MTMA identifies significant taxa-disease associations while accounting for concordance in the direction and variance of the effect across cohorts allowing for a more comprehensive view of the microbiome’s role in disease.

### Multi-technology meta-analysis identifies concordant strain signatures that are supported by multiple isolated analyses which often fail to identify these changes as significant

Unlike significant taxa identified by isolated datasets were trends in the association were often not supported across cohorts (Supplementary Figure 1), significant associations identified by MTMA are driven by the directional concordance of the strain and supported by multiple cohorts (Figure 4 and Supplementary Figures 2–5). In fact, we observed that for the majority of these strains association with homeostasis (green) or dysbiosis (pink) was supported by all datasets the strain was detected in demonstrating that the significantly DA strains identified by MTMA reveal perturbations that are consistent across cohorts and DNA-profiling technologies. In a few cases where there was discordance in trends (example, any strain represented by a large point in the left panel of Supplementary Figure 3) these could be attributed to isolated datasets that had low confidence in the direction of association of a strain (dotted lines). Several strain-disease associations identified in MTMA were not identified in isolated analyses of the datasets (Figure 4 and Supplementary Figures 2–5; orange dots). This could likely be due to isolated studies individually being underpowered to detect this change given the lack of confidence in the direction of association inferred in many of these isolated datasets (dotted lines). In these cases, MTMA inferred a significant association by integrating the observed effects and associated variances across datasets. Thus, MTMA corroborated findings from isolated analysis if supported concordantly across datasets but eliminates discordant and identified novel disease-strain associations that isolated analyses failed to detect. Furthermore, several associations uncovered herein were from the integration of cohorts characterized by different DNA-profiling technologies which would not have been possible without a multi-technology integration approach.

### Multi-technology meta-analysis identifies novel dysbiosis and homeostasis-associated species and strains and confirms previously established taxa associated with Crohn’s disease and Ulcerative colitis

Given that few studies have investigated the microbiome in both stool and mucosa in an IBD patient population (Altomare et al., 2019; Lo Presti et al., 2019), we also used MTMA to identify cross-cohort microbiome associations that were specific to or common across microbial ecosystems represented by stool and mucosal samples. Overall, compared to stool we observed fewer species and strains that were significantly DA in the mucosa. Furthermore, the largest DA species and strains (both in terms of magnitude and significance) were observed in the comparison of stool from control and CD patients (Supplementary Figures 6A,B), indicating that microbiome dysbiosis is both greater and more consistent across cohorts in CD. We also observed that the decrease of several homeostasis-associated bacteria was supported by a greater number of cohorts than an enrichment of dysbiosis-associated bacteria in both stool and mucosa (Supplementary Figure 6C and findings in the top-right quadrant of volcano plots are supported by more cohorts compared to top-left quadrant). Together, these findings point to greater therapeutic potential in targeting the microbiome *via* restoration of missing homeostasis-associated bacteria or functions mediated by these bacteria, especially in CD.

Using MTMA, we were able to confirm the previously observed decrease in certain homeostasis-associated species in the stool of patients with both UC and CD (Figure 5, blue highlights; Supplementary Figure 8) (McIlroy et al., 2018; Schirmer et al., 2019). Other microbiome-IBD associations, that were previously reported in the literature, were only confirmed as significant associations across CD cohorts (Figure 5, red highlights) although non-significant trends were observed across UC cohorts. Further, while the previously reported decrease of *Faecalibacterium prausnitzii* in patients with CD was observed in stool samples, this association was not significant in mucosal tissue (Walters et al., 2014; Schirmer et al., 2019). We identified several taxa that were increased in both the stool and mucosa of control compared to patients with CD and a few that were decreased, such as *Morganella morganii* (Figure 5, purple dots; Supplementary Figures 7, 9). Such microbial changes that are observed in both luminal and mucosal environments could be indicative of systemic perturbation to the gut-microbiome. We also found lower levels of yet-to-be-named species and strains from the *Lachnospiraceae* family in mucosa and stool samples from patients with UC and CD (Supplementary Figures 7–10), presenting novel associations of these taxa in IBD. In addition to confirming the cross-cohort generalizability of previously reported taxa-disease associations in IBD, our MTMA approach identified novel and specific strain-associations within these species presenting genomic targets that can be further interrogated for their functional role in disease.

### Strain-level multi-technology meta-analysis reveals unique taxa-disease associations that are not always recapitulated at the species-level

When comparing MTMA results at the species and strain level, we observed that not all significant strain associations resulted in species-level significance (bottom panels; Figure 5 and Supplementary Figures 7–10) and conversely significant species associations with the disease were not always re-captured at the strain level (gray bars; Figure 5 and Supplementary Figures 7–10). In many cases where significant species association was identified, we observed that only a subset of strains within this species demonstrated significant association with disease (Figure 6 and Supplementary Figure 11). More importantly, we observed that within a species, some strains demonstrated enrichment in disease while others were enriched in control subjects. For example, while the species-level analysis and previous studies reported a decrease in *Odoribacter splanchnicus* in patients with CD (Schirmer et al., 2019), we observed both significant disease and homeostasis-associated strains within this species (Figure 6, fourth taxa from the right). To gain insight into the functional differences between the disease and homeostasis-associated *O. splanchnicus* strains, we compared the annotated KEGG pathway profiles of these strains’ genomes and observed greater coverage of a D-alanine metabolism pathway (path:map00473; Supplementary Figure 12) in the homeostasis-associated strain (t__266395). Alanine is reported to reduce experimental liver damage by a direct effect on hepatocytes (Maezono et al., 1996). The ability to modulate alanine metabolism by t__266395 may confer a beneficial effect on the liver, which is known to be associated with gut mucosal immunity (Trivedi and Adams, 2016). These findings together highlight that strain identity even within species may be important to the role played by a bacterium in disease.

### Multi-technology meta-analysis uncovers strains that demonstrate cross-cohort association with inflammatory bowel disease, including across disease subtypes and gut-microbial ecosystems, presenting novel therapeutic and diagnostic opportunities

Strains that are significantly enriched or decreased in UC and CD subjects in both the stool and mucosa may point to an IBD biology that influences both disease subtypes and to potential drivers of systemic gut dysbiosis. A total of 267 of the 305 strains identified as significantly DA by MTMA were changed specifically to a disease subtype and an ecosystem (Supplementary Figure 13A). Among strains that demonstrated significant associations in both UC and CD subjects compared to controls, only one strain, *Gemminger formicilis* t__83170, was identified as significantly decreased in both luminal (stool) and intestinal-mucosa from UC and CD subjects (Supplementary Figure 13B). Identification of strain signatures that were decreased in both UC and CD patients across cohorts motivated us to examine taxa associations with IBD agnostic of disease subtype or microbial ecosystem. A total of 329 strains were detected in at least one dataset comparing UC and CD to control subjects in both mucosa and stool. An MTMA integrating isolated-dataset results across all contrasts for these strains identified 40 strains that demonstrated cross-cohort associations with IBD in both the luminal and mucosal ecosystems and both disease subtypes. More strains were consistently decreased in IBD patients compared to strains that were consistently decreased in control subjects across cohorts (Supplementary Figure 14). This finding is consistent with observations of a decrease of homeostasis-associated genera across IBD cohorts by Duvallet et al. (2017), re-emphasizing a need for therapeutic strategies in IBD that target remediation of decreased homeostasis-associated strains.

Of particular interest were four strains (*A. putredenis* t__80573, *G. formicilis* t__83170, and two *Lachnospiraceae* strains t__256727 and t__133679) that were significantly enriched and one strain (*D. invisus* t__28462) that was decreased in controls compared to case subjects. The association of these strains with the disease was supported by > 12 cohorts and also supported in both stool and mucosa (Figure 7). Significant associations for a few of these strains were consistent with the literature. A decrease of *A. putredinis* and *G. formicilis* in patients with IBD has been reported previously (de Meij et al., 2018; Kowalska-Duplaga et al., 2019; Schirmer et al., 2019). A study characterizing the microbiome using a molecular fingerprinting technique reported a decrease of *D. invisus* in patients with CD (Joossens et al., 2011) while our MTMA strain-level integration of multiple IBD cohorts demonstrated a significant decrease of the *D. invisus* strain t__28462 in controls compared to patients with IBD. We also identified the enrichment of two *Lachnospiraceae* strains across control compared to patients with IBD in multiple cohorts that are yet-to-be-named even at the genus level. The enrichment of this strain in control subjects was supported by datasets using multiple DNA-profiling technologies (Figure 7; point shapes) further increasing the confidence in the identified association of these strains. While a decrease of *Lachnospiraceae* in IBD patients has been presented in multiple studies (Schirmer et al., 2019), none described these specific strains.

**FIGURE 8:**
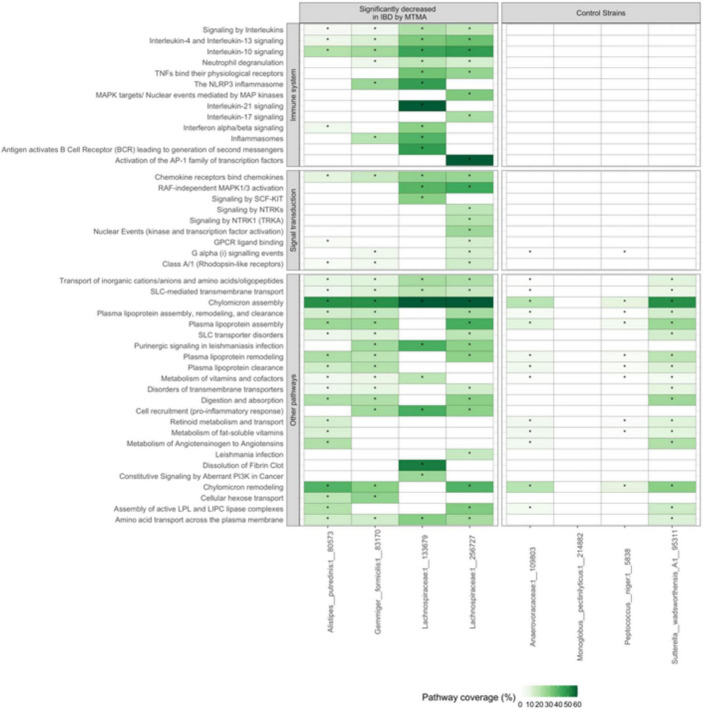
Strains that are ubiquitously decreased in patients with IBD are associated with modulation of immune and signal-transduction related pathways. Gene-expression patterns correlating with the abundance of four strains identified as significantly enriched in controls compared to patients with IBD by MTMA **(left panel)** and four strains that were not significant in this MTMA **(right panel)** were determined. Pathway enrichment analysis was used to identify pathways that were significantly enriched in the gene-expression pattern associated with each strain. Pathway coverage across the IBD and control strains are shown. Light to dark green indicates higher pathway coverage. White cells without an * indicate that the pathway was not significantly enriched in the gene-expression pattern associated with that strain. Only the top 20 pathways that were significantly enriched in at least one of the IBD strains are shown. Significance was determined at adjusted *p* < 0.2.

To gain insight into potential disease-modulating functions of the four strains enriched in control compared to patients with IBD we examined host gene-expression patterns correlating with the abundance of these strains in a cohort of IBD patients. A total of 140 patients (15 controls, 77 UC, 48 CD) in SG-Cohort-1 had both microbiome (PhyloChip) and host-gene expression profiles (RNAseq) of their mucosal samples available for analysis. Gene-expression patterns indicated significant enrichment of immune-related and signal-transduction pathways associated with these four strains (Figure 8). For the two *Lachnospiraceae* strains, we observed significant enrichment of multiple inflammation-related pathways such as those involved in interleukin-signaling. Many of these pathways, including TNF-α, IL-17, and IL-10 signaling, are known target therapeutic mechanisms in IBD (Weisshof et al., 2018; Giuffrida et al., 2019). To determine if enrichment of immune-related pathways was specific to the strains we identified herein, we compared pathways enriched in correlation with the four strains to those enriched in correlation with strains that were not significantly associated with IBD in this MTMA (strains that are neither consistently enriched nor decreased across IBD cohorts; control strains). Most of the immune-related and signal-transduction pathways were not associated with the control strains indicating that enrichment of these known IBD-related pathways is specific to the strains we identified here as decreased in patients with IBD (Figure 8). This analysis points to the value of the MTMA approach that identifies specific strains associated with the disease across cohorts and hence enables insight into the biological relevance of these strains in a disease context.

## Discussion

It is apparent from our re-analysis of published datasets that there exist significant variations across cohorts, even when datasets are analyzed through a standardized pipeline (Figure 2 and Supplementary Figure 1). MTMA enabled the synthesis of existing knowledge of the microbiome in IBD and uncovered previously unreported perturbations in IBD that are applicable across geographically dispersed cohorts overcoming limitations of previous meta-analyses that were restricted in their view of the microbiome in disease to that offered by one DNA-profiling technology. Combining datasets profiled using different technologies not only allowed us to view the microbiome in IBD through a comprehensive lens but also increased confidence in strain-disease associations that were supported by multiple technologies, especially in cases where novel associations with unnamed strains were identified (*Lachnospiraceae* strains; Figure 7). We acknowledge that even with this MTMA approach, the taxa-disease associations identified herein is subject to biases from differences between isolated studies in terms of experimental, sequencing, and cohort demographics. However, by focusing on associations that are supported by multiple cohorts, MTMA limits the identification of spurious associations that may arise from experimental biases in the analysis of a single cohort or a meta-analysis integrating data profiled on one technology one.

While the analysis at higher-order taxonomic levels of the microbiome provides valuable insight, our comparative MTMA at the species and strain level clearly demonstrates strain-specific associations with disease or health even within a species. Strain-specific variations in the metabolic capabilities of bacterial species have long been established but strain-level variations within the human microbiome are only recently being explored (Yan et al., 2020). We attempted to characterize functional differences between strains identified here as disease or homeostasis-associated. However, we observed that the majority of their functions remain to be elucidated with on average, over 70% of the predicted genes from a strain’s genome having no known KEGG orthologs. This necessitates improved functional annotation of proteins derived from the human microbiome. Recent work into deciphering the functional annotation for small proteins derived from human-associated metagenomes has been an important step in this direction (Sberro et al., 2019). In particular, little is known about the two *Lachnospiraceae* strains that are decreased in IBD stools and mucosa, besides their genome assemblies. Just as NIH projects such as “Most Wanted” identified abundant 16S rRNA amplicons that inspired a search for their genomes of origin (Fodor et al., 2012), we believe a new project is needed to elucidate the phenotypes of strains with known genomes and highly significant associations with disease and health. The *Lachnospiraceae* strains pinpointed by MTMA are excellent candidates for functional studies that characterize how peptides, proteins, or metabolites from these strains interact with human cells to begin to unravel their role in IBD.

Our approach identified novel species and strains that were differentially abundant in patients with IBD compared to control subjects and confirmed previously published findings such as a decrease of *Faecalibacterium prausnitzii* in patients with CD (Schirmer et al., 2019). Furthermore, in many cases for the first time identified specific strains and the applicability of these findings across cohorts. Overall, we observed greater support across cohorts for species and strains that were decreased in patients with UC or CD compared to controls (Supplementary Figure 6), pointing to a greater therapeutic potential of targeting the microbiome by restoring missing homeostasis-associated bacteria or their associated functions. We also observed that MTMA identified strain associations specific to UC or CD, implying potential distinct microbial drivers of the two disease subtypes. Particularly, in comparing stool samples between CD and control patients, we observed many significant and large taxa-disease associations (Supplementary Figure 6; larger values of significance and log-2 fold change), indicating that drivers of microbial dysbiosis are likely larger and consistent across patients with CD as compared to UC. Alternatively, this finding could point to patient stratification within patients with UC or other confounders that elude the identification of strong cross-cohort microbial drivers of UC. Our integrated analysis combining information across disease subtypes and microbial ecosystems revealed for the first time four strains that were consistently decreased in IBD in both the gut lumen and mucosa. These strains may represent a starting point for the development of therapeutic interventions targeting restoration of these strains or their functions to levels observed in control subjects. Furthermore, signatures that are reproducible across cohorts, ecosystems, disease subtypes, and DNA profiling technologies can present opportunities for the development of a ubiquitous IBD-strain biomarker. Our effort to understand how these strains interact with the host at a mechanistic level, revealed enrichment of inflammation-related pathways in host-gene expression correlating with the two *Lachnospiraceae* strains that were decreased in IBD. While this analysis points to potential pathways *via* which these strains could confer a host benefit in IBD, further experimental and omics analyses are required to understand how these strains interact with the host.

Multi-technology meta-analysis reveals novel and previously unpublished species and strains that are enriched or decreased in IBD compared to control patients from a systematic re-analysis and integration of existing public and new datasets. Our comparative analysis at the species and strain level highlights the importance of strain-specific association with disease or health even within a species and underscores the need for fine-grained taxonomic analysis of the microbiome to generate testable hypotheses and disease-specific therapeutic strategies. We believe applying the MTMA framework, with its ability to integrate a growing number of datasets across DNA profiling technologies and pinpoint specific strains, will allow for the identification of robust microbiome modulators of disease.

## Data availability statement

The datasets presented in this study can be found in online repositories. The names of the repository/repositories and accession number(s) can be found below: https://www.ncbi.nlm.nih.gov/, PRJNA398187 and PRJNA527097.

## Author contributions

JR contributed to the conception and design of work, acquisition, analysis and interpretation of data, and drafted and substantively revised the manuscript. ER contributed to the acquisition of data. C-EC, GK, PB, and MC contributed to the analysis and interpretation of data, and revision of the manuscript. NN contributed to the analysis of data. AH contributed to the design of work, acquisition, analysis and interpretation of data, and substantively revised the manuscript. SI and TD contributed to the conception and design of work, acquisition, analysis and interpretation of data, and substantively revised the manuscript. KD contributed to the conception and design of work, interpretation of data, and substantively revised the manuscript. All authors contributed to the article and approved the submitted version.

## Footnotes

1
http://strainselect.secondgenome.com

2
https://github.com/FrederickHuangLin/ANCOM

## Supplementary material

Supplementary Figure 1 Significant findings from isolated dataset analysis often do not exhibit concordance in the direction of differential abundance across multiple datasets. Left panel tallies the number of significant strains (*adjusted p* < *0.01*) in each isolated dataset with sub-tallies by enrichment (green) or decrease (pink) in control compared to case subjects as defined by the direction of log-2 fold change. In the middle and right panels, log-2 fold changes in each isolated dataset are shown for strains that are significantly DA in at least one isolated dataset and detected in at least two isolated datasets. Closed and open triangles indicate significant and non-significant findings in each isolated dataset, respectively. Strains are grouped by concordance in direction of differential abundance across all datasets or the lack thereof. Panels **(a,b)** summarize findings from analysis of mucosa from each isolated dataset in comparison of control to UC and CD subjects, respectively. Similarly, panels **(c,d)** summarize findings for analysis of stool from controls compared to UC and CD subjects, respectively.

Supplementary Figure 2 Differentially abundant strains in Crohn’s disease identified herein are concordant across cohorts (only adjusted *p* < 0.01 and > 0.0001 are shown). Strains that are significantly enriched **(a)** or decreased **(b)** in stool from controls compared to CD subjects based on strain-level MTMA. Strains (dots) are sized by the number of isolated datasets the strain is detected and colored as follows: significant by 1 or more isolated analysis and MTMA (blue) or MTMA only (orange). Strains are connected to isolated datasets (gray squares) they are detected in. Line color indicates enrichment (green) or decrease (pink) of strains in controls compared to case subjects in each isolated dataset. Thick and thin lines indicate significant and non-significant findings in isolated datasets, respectively. Solid and dashed lines correspond to isolated datasets that are confident or not-confident in the direction of the log-2 fold change, respectively. Confidence is determined as cases where the lower and upper bounds of the 95% confidence interval associated with a log-2 fold change are in the same direction as the log-2 fold change.

Supplementary Figure 3 Differentially abundant strains in Ulcerative Colitis subjects identified herein are concordant across cohorts in mucosa samples. **Left** and **right** panels plot strains that are significantly enriched or decreased in controls compared to Ulcerative colitis subjects in mucosa samples based on strain-level MTMA, respectively. Strains (dots) are sized by the number of isolated datasets a strain is detected and colored as follows: significant by 1 or more isolated analysis and MTMA (blue) or MTMA only (orange). Strains are connected to isolated datasets (gray squares) they are detected in. Line color indicates enrichment (green) or decrease (pink) of strains in controls compared to case subjects in each isolated dataset. Thick and thin lines indicate significant and non-significant findings in isolated datasets, respectively. Solid and dashed lines correspond to isolated datasets that are confident or not-confident in the direction of the log-2 fold change, respectively. Confidence is determined as cases where the lower and upper bounds of the 95% confidence interval associated with a log-2 fold change are in the same direction as the log-2 fold change. Significance was determined at an *adjusted p* < *0.01*.

Supplementary Figure 4 Differentially abundant strains in Crohn’s disease subjects identified herein are concordant across cohorts in mucosa samples. **Left** and **right** panels plot strains that are significantly enriched or decreased in controls compared to Crohn’s disease subjects in mucosa samples based on strain-level MTMA, respectively. Strains (dots) are sized by the number of isolated datasets a strain is detected and colored as follows: significant by 1 or more isolated analyses and MTMA (blue) or MTMA only (orange). Strains are connected to isolated datasets (gray squares) they are detected in. Line color indicates enrichment (green) or decrease (pink) of strains in control compared to case subjects in each isolated dataset. Thick and thin lines indicate significant and non-significant findings in isolated datasets, respectively. Solid and dashed lines correspond to isolated datasets that are confident or not-confident in the direction of the log-2 fold change, respectively. Confidence is determined as cases where the lower and upper bounds of the 95% confidence interval associated with a log-2 fold change are in the same direction of the log-2 fold change. Significance was determined at an *adjusted p* < *0.01*.

Supplementary Figure 5 Differentially abundant strains in Ulcerative Colitis subjects identified herein are concordant across cohorts in stool samples. **Left** and **right** panels plot strains that are significantly enriched or decreased in controls compared to Ulcerative colitis subjects in stool samples based on strain-level MTMA, respectively. Strains (dots) are sized by the number of isolated datasets a strain is detected and colored as follows: significant by 1 or more isolated analysis and MTMA (blue) or MTMA only (orange). Strains are connected to isolated datasets (gray squares) they are detected in. Line color indicates enrichment (green) or decrease (pink) of strains in controls compared to case subjects in each isolated dataset. Thick and thin lines indicate significant and non-significant findings in isolated datasets, respectively. Solid and dashed lines correspond to isolated datasets that are confident or not-confident in the direction of the log-2 fold change, respectively. Confidence is determined as cases where the lower and upper bounds of the 95% confidence interval associated with a log-2 fold change are in the same direction of the log-2 fold change. Significance was determined at an *adjusted p-value cutoff of 0.01*.

Supplementary Figure 6 Multi-technology meta-analysis identifies greater number of significantly differentially abundant species and strains in stool compared to mucosa and in Crohn’s disease as compared to ulcerative colitis. MTMA-derived adjusted *p*-values and log-2 fold changes are plotted. Data points are colored according to the number of cohorts in which a species or strain was detected and shaped by the number of DNA-profiling technologies datasets integrated into MTMA were characterized. The red-dashed line corresponds to an *adjusted p-value of 0.01.* Species or strains significantly increased in control subjects plot in the upper right quadrant, whereas those decreased in control subjects plot in the upper left quadrant. Panels **(a,b)** correspond to species and strain-level findings, respectively. **(c)** The average number of cohorts in which a significant DA strain or species is detected and plotted. Green and pink represent the number of cohorts supporting strains that are significantly increased and decreased in controls as compared to case subjects, respectively.

Supplementary Figure 7 Multi-technology meta-analysis identifies species and strains that are yet to be named even at the species-level that are differentially abundant in stool samples from Crohn’s as compared to control subjects. Left panel plots the log-2 fold change of differential abundance aggregated at the species level. Bars are colored by the number of strains within a species that are significantly DA with gray bars indicating cases where only species-level significance is observed. Right strip indicates phylum-level placement of each species. Top panel plots cases with species-level significant differences with or without strain-level significant differences. Bottom panel plots cases with only strain-level but no species-level significant differences. Significance was determined at *adjusted p* < *0.01*. Purple dots point to species decreased in both stool and mucosa of controls compared to CD patients. Only species that are not taxonomically named are shown.

Supplementary Figure 8 Novel dysbiosis and homeostasis-associated species and strains in the gut lumen of Ulcerative colitis subjects that were previously unreported in isolated analyses. Left panel plots the log-2 fold change of differential abundance aggregated at the species level. Bars are colored by the number of strains within a species that are significantly differentially abundant with gray bars indicating cases where only species-level significance is observed. Right strip indicates phylum-level placement of each species. Top panel plots cases with species-level significant differences with or without strain-level significant differences. Bottom panel plots cases with only strain-level but no species-level significant differences. Significance was determined at *adjusted p* < *0.01*.

Supplementary Figure 9 Novel dysbiosis and homeostasis-associated species and strains in the mucosa of Crohn’s disease subjects that were previously unreported in isolated analyses. Left panel plots the log-2 fold change of differential abundance aggregated at the species level. Bars are colored by the number of strains within a species that are significantly differentially abundant with gray bars indicating cases where only species-level significance is observed. Right strip indicates phylum-level placement of each species. Top panel plots cases with species-level significant differences with or without strain-level significant differences. Bottom panel plots cases with only strain-level but no species-level significant differences. Significance was determined at *adjusted p* < *0.01*.

Supplementary Figure 10 Novel dysbiosis and homeostasis-associated species and strains in the mucosa of Ulcerative Colitis subjects that were previously unreported in isolated analyses. Left panel plots the log-2 fold change of differential abundance aggregated at the species level. Bars are colored by the number of strains within a species that are significantly differentially abundant with gray bars indicating cases where only species-level significance is observed. Right strip indicates phylum-level placement of each species. Top panel plots cases with species-level significant differences with or without strain-level significant differences. Bottom panel plots cases with only strain-level but no species-level significant differences. Significance was determined at *adjusted p* < *0.01*.

Supplementary Figure 11 Strain-identity even within a species is important to the functional role of a bacterium in IBD. Top strip plots enrichment (green) or decrease (pink) of species in controls as compared to case subjects. Bottom panel plots the log-2 fold change of strains within a species with closed and open triangles indicating significant and non-significant findings in *strain*-level MTMA, respectively. Significance was determined at an *adjusted p* < *0.01*. Panels **(a)** through **(c)** summarize findings for controls compared to UC in mucosa, controls compared to CD in mucosa, controls compared to UC in stool, respectively.

Supplementary Figure 12 Strains within a species demonstrate differences in their functional potential. KEGG-pathway profiles of two *Odoribacter splanchnicus* strains that were identified as significantly enriched (t__237969) and decreased (t__266395) in stool from CD as compared to control subjects by MTMA are shown. Color gradient in the heat map represents the percent coverage of known genes in the pathway that are identified in the genome of the strain. All pathways supported by the presence of at least 20% of the known genes constituting the pathway in one of the strains are shown.

Supplementary Figure 13 Multi-technology meta-analysis identifies strain-associations that are often specific to a disease subtype and microbial ecosystem with few strains that demonstrate significant associations in multiple MTMAs. **(a)** Number of strains that are significantly DA by MTMA in each of the two subtypes (Controls compared to UC or CD) for each gut-microbial ecosystem (stool/luminal and mucosa) with sub-tallies colored according to the number of MTMAs a strain is significant. **(b)** MTMA-derived log-2 fold changes for strains identified as significantly (denoted by asterisks) DA in two or more contrasts by MTMA are shown. Significance was determined at *adjusted p* < *0.01.*

Supplementary Figure 14 Multi-technology meta-analysis identifies greater number of homeostasis as compared to dysbiosis-associated strains in IBD that are consistently decreased across disease subtypes, gut-microbial ecosystems, and DNA-profiling technologies. MTMA-derived adjusted *p*-values and log-2 fold changes are plotted from an MTMA integrating all IBD datasets described here in. Data points are colored according to the proportion of comparisons in which a strain demonstrated concordance in direction of log-2 fold change and shaped by the DNA-profiling technologies used for characterization of the datasets. The red-dashed line corresponds to an *adjusted p-value of 0.01.* Strains significantly enriched in controls compared to IBD subjects plot in the upper right quadrant, whereas those decreased in control subjects plot in the upper left quadrant.

Supplementary Figure 15 Multi-technology meta-analysis identifies strains that are consistently associated (enriched or decreased) with IBD across disease subtypes, gut-microbial ecosystems, cohorts and DNA-profiling technologies. Each panel plots log-2 fold change from MTMA and isolated datasets for strains that are significantly DA in an MTMA integrating all datasets and comparisons described here in. For isolated datasets, points are shaped by the DNA-profiling technology used to characterize the microbiome and colored by gut-microbial ecosystems and disease subtypes being compared to controls. Error bars correspond to the 95% confidence interval. Significance was determined at an *adjusted p* < *0.01*. Plots summarizing results for strains that are detected in less than 75% of the contrasts integrated into the MTMA are shown here.

Supplementary Table 1 Details pertaining to cohorts, datasets, and comparisons analyzed.

Supplementary Table 2 Examples of 16S rRNA gene sequences observed in clinical subjects, their identities to publicly available reference genes from known isolated strains, and interpretations of which strain was selected, if any, as the best unique match. Two cases (Examples 1 and 2) where a strain is not selected from a clinical sequence are shown followed by three cases (Examples 3–5) where a strain is selected. Displayed beneath each ASV are the reference genes with identity from highest downward to the first gene or tied genes below 99% identity. Reference genes are indicated by NCBI accession number followed by a colon, the nucleotide span, and the strand in parentheses. Since one strain can have genes deposited from multiple DNA sequencing institutes and have various identifiers in international culture collections, synonymous terms for the same strain are included for convenience. The StrainSelect ID is the single identifier representing the entire set of synonyms. A sequence was assigned to a strain only when it met two conditions: (1) it matches at least one reference gene from one strain with ≥ 99% identity, and (2) the highest identity matches to any gene from a different strain is less than the top strain.

Supplementary Table 3 Species identified as significantly differentially enriched or decreased in UC or CD compared to control subjects by MTMA. Significance was determined at *adjusted p* < *0.01.* Log-2 fold change of case over control in comparison of UC or CD to control subjects in stool or mucosa samples are tabulated. Negative and positive log-2 fold changes correspond to species decreased or enriched in controls compared to case subjects, respectively.

Supplementary Table 4 Strains identified as significantly differentially enriched or decreased in UC or CD compared to control subjects by MTMA. Significance was determined at *adjusted p* < *0.01.* Log-2 fold change of case over control in comparison of UC or CD to control subjects in stool or mucosa samples are tabulated. Negative and positive log-2 fold changes correspond to strains decreased or enriched in controls compared to case subjects, respectively.
