# Supplementary figures and images for: Strain level and comprehensive microbiome analysis in inflammatory bowel disease via multi-technology meta-analysis identifies key bacterial influencers of disease
Source: Front Microbiol. 2022 Oct 14;13:961020. doi: 10.3389/fmicb.2022.961020 (PMC9614153; doi:10.3389/fmicb.2022.961020)

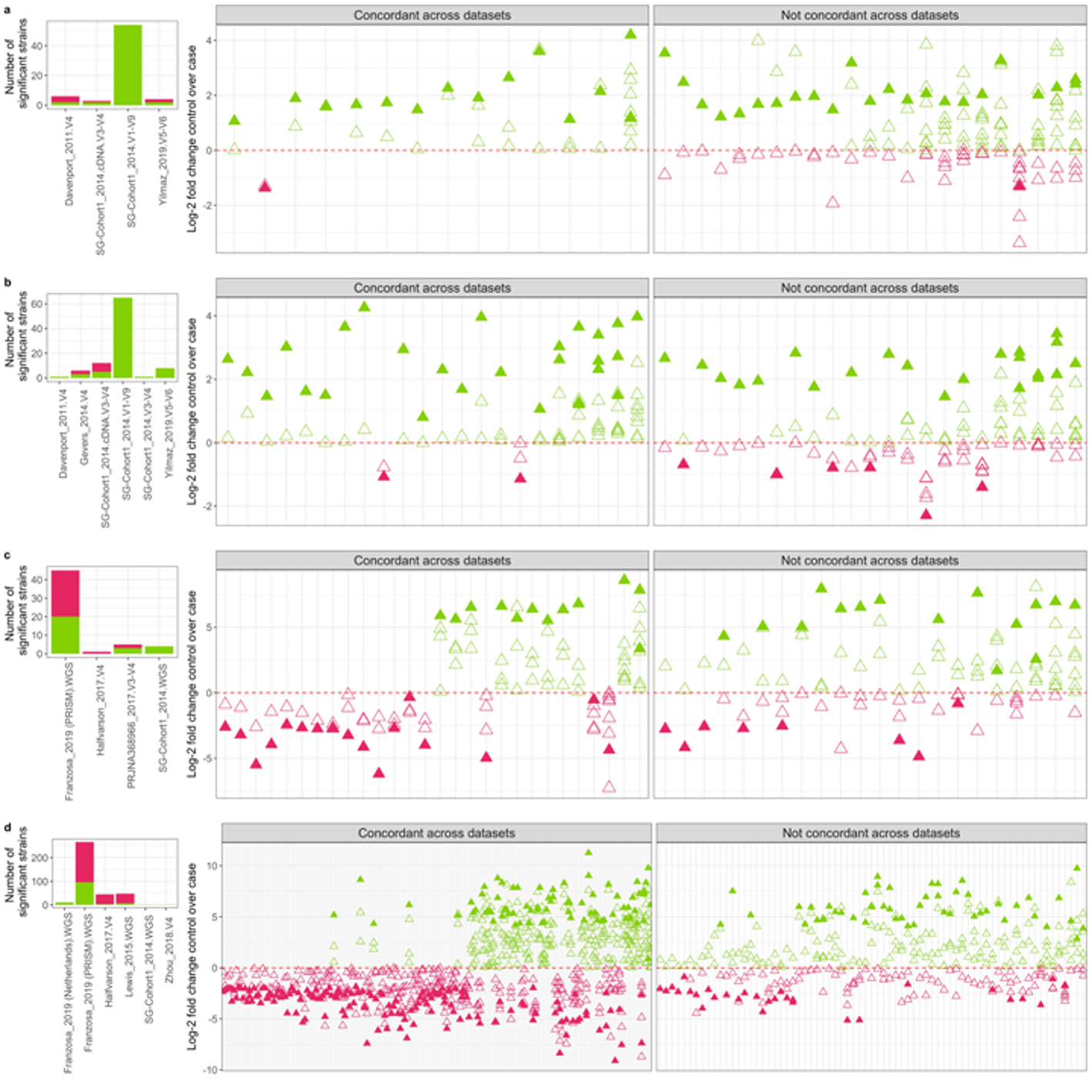

Supplement: Supplementary Figure 1 — Significant findings from isolated dataset analysis often do not exhibit concordance in the direction of differential abundance across multiple datasets. Left panel tallies the number of significant strains (adjusted p < 0.01) in each isolated dataset with sub-tallies by enrichment (green) or decrease (pink) in control compared to case subjects as defined by the direction of log-2 fold change. In the middle and right panels, log-2 fold changes in each isolated dataset are shown for strains that are significantly DA in at least one isolated dataset and detected in at least two isolated datasets. Closed and open triangles indicate significant and non-significant findings in each isolated dataset, respectively. Strains are grouped by concordance in direction of differential abundance across all datasets or the lack thereof. Panels (a,b) summarize findings from analysis of mucosa from each isolated dataset in comparison of control to UC and CD subjects, respectively. Similarly, panels (c,d) summarize findings for analysis of stool from controls compared to UC and CD subjects, respectively. [file Image_1.TIFF]

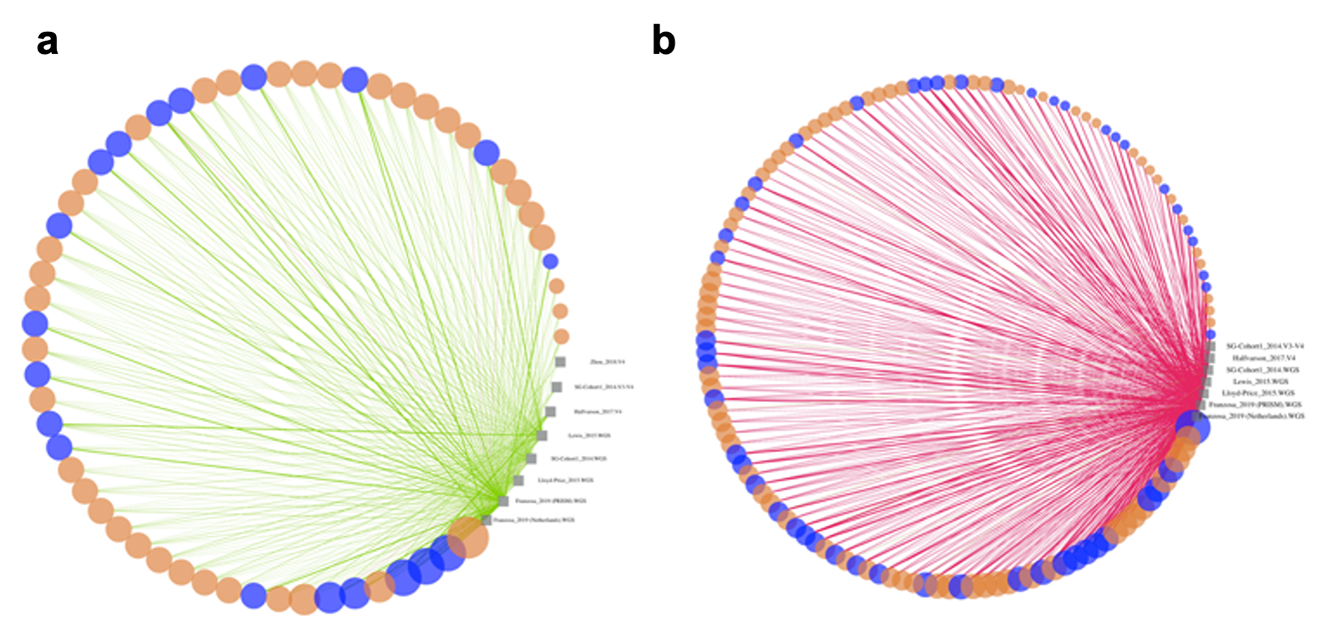

Supplement: Supplementary Figure 2 — Differentially abundant strains in Crohn’s disease identified herein are concordant across cohorts (only adjusted p < 0.01 and > 0.0001 are shown). Strains that are significantly enriched (a) or decreased (b) in stool from controls compared to CD subjects based on strain-level MTMA. Strains (dots) are sized by the number of isolated datasets the strain is detected and colored as follows: significant by 1 or more isolated analysis and MTMA (blue) or MTMA only (orange). Strains are connected to isolated datasets (gray squares) they are detected in. Line color indicates enrichment (green) or decrease (pink) of strains in controls compared to case subjects in each isolated dataset. Thick and thin lines indicate significant and non-significant findings in isolated datasets, respectively. Solid and dashed lines correspond to isolated datasets that are confident or not-confident in the direction of the log-2 fold change, respectively. Confidence is determined as cases where the lower and upper bounds of the 95% confidence interval associated with a log-2 fold change are in the same direction as the log-2 fold change. [file Image_2.TIFF]

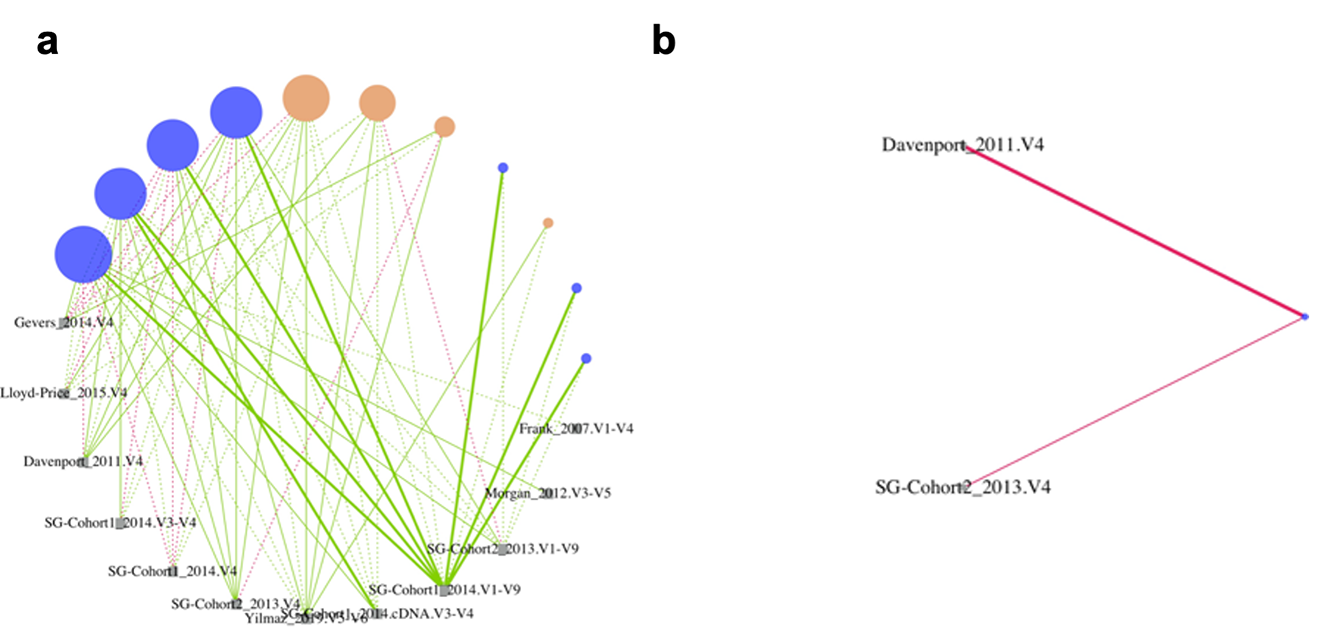

Supplement: Supplementary Figure 3 — Differentially abundant strains in Ulcerative Colitis subjects identified herein are concordant across cohorts in mucosa samples. Left and right panels plot strains that are significantly enriched or decreased in controls compared to Ulcerative colitis subjects in mucosa samples based on strain-level MTMA, respectively. Strains (dots) are sized by the number of isolated datasets a strain is detected and colored as follows: significant by 1 or more isolated analysis and MTMA (blue) or MTMA only (orange). Strains are connected to isolated datasets (gray squares) they are detected in. Line color indicates enrichment (green) or decrease (pink) of strains in controls compared to case subjects in each isolated dataset. Thick and thin lines indicate significant and non-significant findings in isolated datasets, respectively. Solid and dashed lines correspond to isolated datasets that are confident or not-confident in the direction of the log-2 fold change, respectively. Confidence is determined as cases where the lower and upper bounds of the 95% confidence interval associated with a log-2 fold change are in the same direction as the log-2 fold change. Significance was determined at an adjusted p < 0.01. [file Image_3.TIFF]

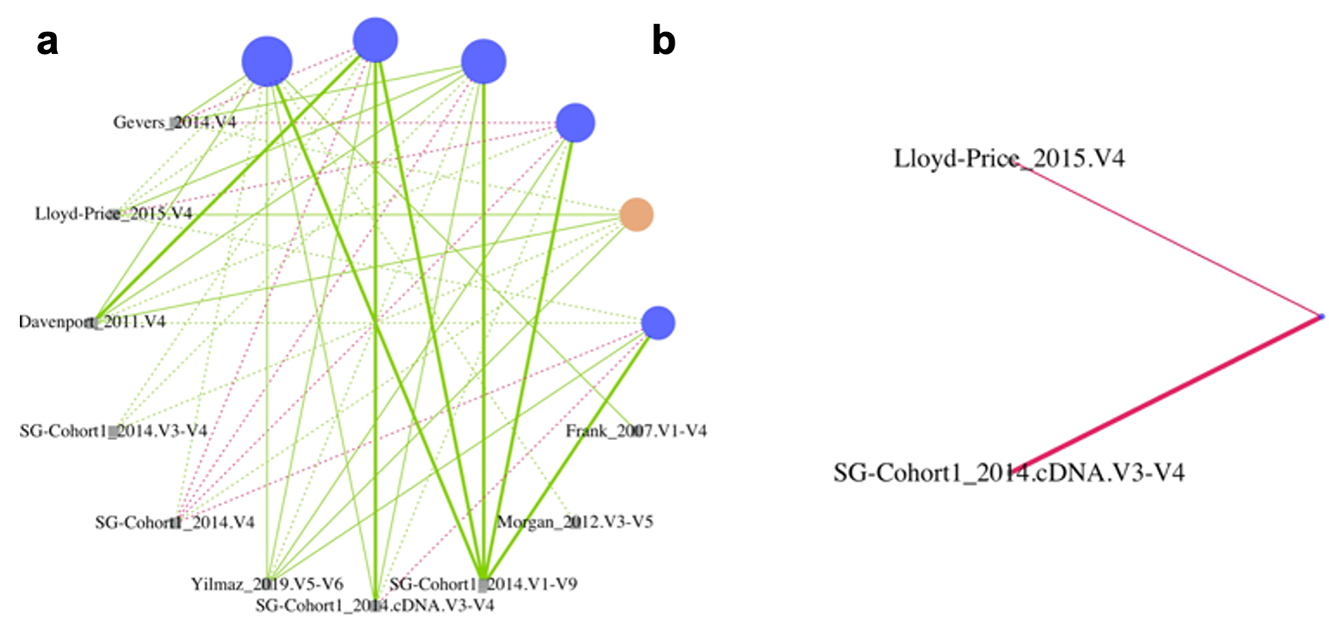

Supplement: Supplementary Figure 4 — Differentially abundant strains in Crohn’s disease subjects identified herein are concordant across cohorts in mucosa samples. Left and right panels plot strains that are significantly enriched or decreased in controls compared to Crohn’s disease subjects in mucosa samples based on strain-level MTMA, respectively. Strains (dots) are sized by the number of isolated datasets a strain is detected and colored as follows: significant by 1 or more isolated analyses and MTMA (blue) or MTMA only (orange). Strains are connected to isolated datasets (gray squares) they are detected in. Line color indicates enrichment (green) or decrease (pink) of strains in control compared to case subjects in each isolated dataset. Thick and thin lines indicate significant and non-significant findings in isolated datasets, respectively. Solid and dashed lines correspond to isolated datasets that are confident or not-confident in the direction of the log-2 fold change, respectively. Confidence is determined as cases where the lower and upper bounds of the 95% confidence interval associated with a log-2 fold change are in the same direction of the log-2 fold change. Significance was determined at an adjusted p < 0.01. [file Image_4.TIFF]

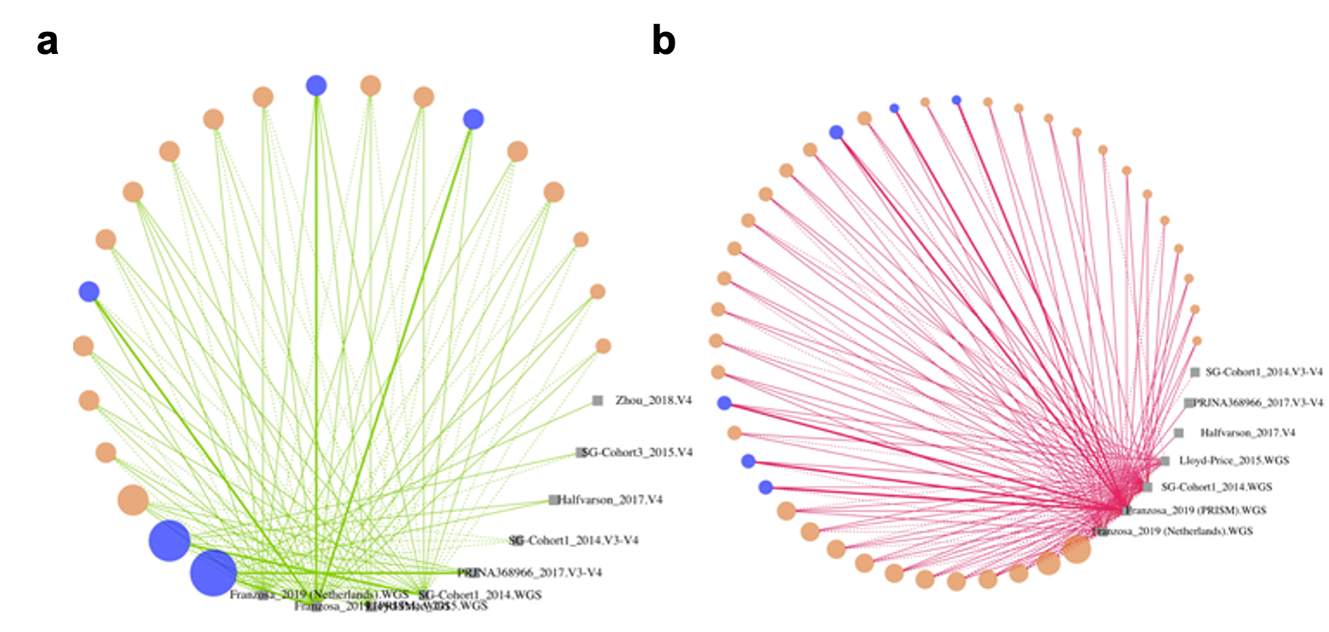

Supplement: Supplementary Figure 5 — Differentially abundant strains in Ulcerative Colitis subjects identified herein are concordant across cohorts in stool samples. Left and right panels plot strains that are significantly enriched or decreased in controls compared to Ulcerative colitis subjects in stool samples based on strain-level MTMA, respectively. Strains (dots) are sized by the number of isolated datasets a strain is detected and colored as follows: significant by 1 or more isolated analysis and MTMA (blue) or MTMA only (orange). Strains are connected to isolated datasets (gray squares) they are detected in. Line color indicates enrichment (green) or decrease (pink) of strains in controls compared to case subjects in each isolated dataset. Thick and thin lines indicate significant and non-significant findings in isolated datasets, respectively. Solid and dashed lines correspond to isolated datasets that are confident or not-confident in the direction of the log-2 fold change, respectively. Confidence is determined as cases where the lower and upper bounds of the 95% confidence interval associated with a log-2 fold change are in the same direction of the log-2 fold change. Significance was determined at an adjusted p-value cutoff of 0.01. [file Image_5.TIFF]

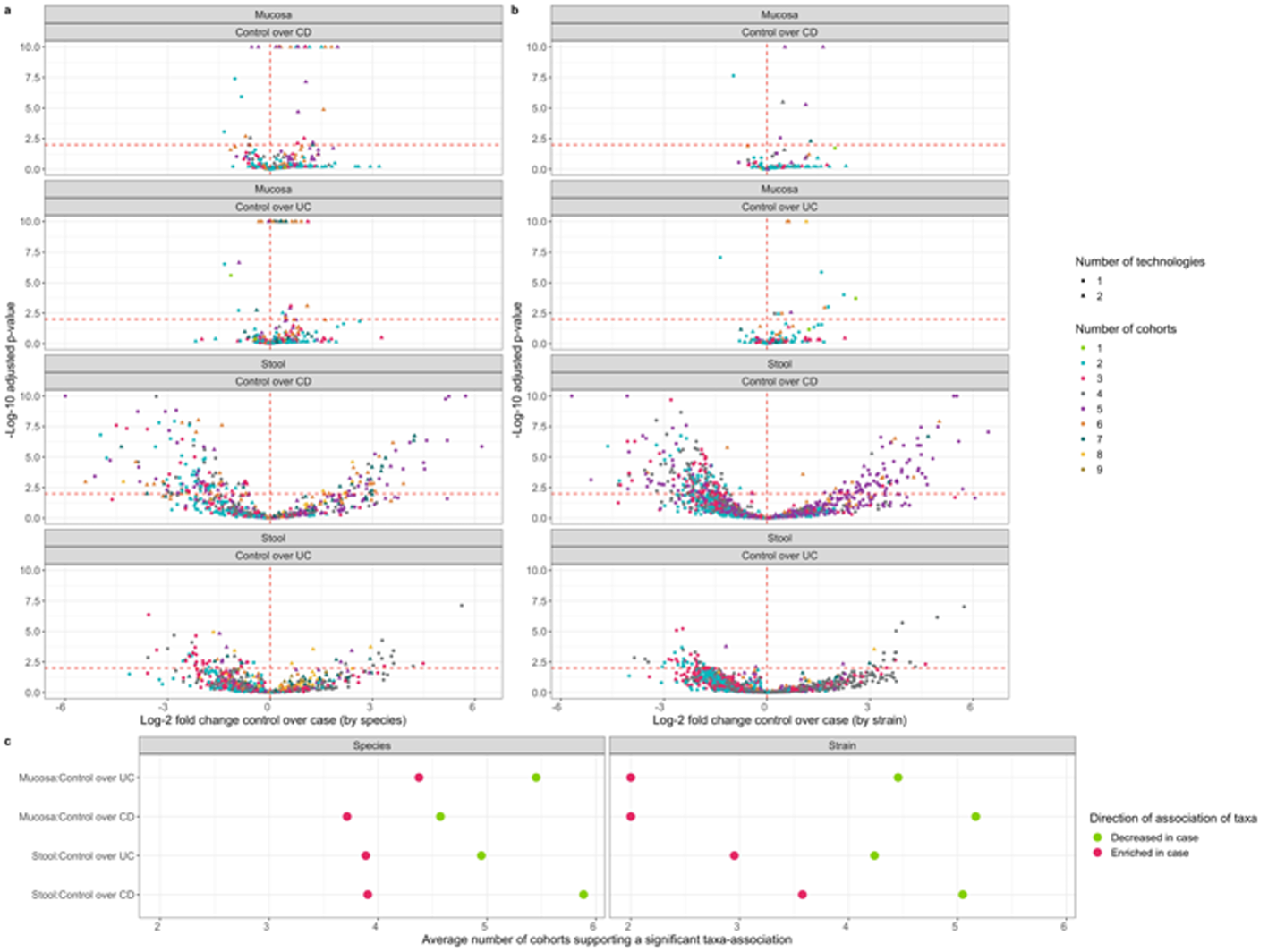

Supplement: Supplementary Figure 6 — Multi-technology meta-analysis identifies greater number of significantly differentially abundant species and strains in stool compared to mucosa and in Crohn’s disease as compared to ulcerative colitis. MTMA-derived adjusted p-values and log-2 fold changes are plotted. Data points are colored according to the number of cohorts in which a species or strain was detected and shaped by the number of DNA-profiling technologies datasets integrated into MTMA were characterized. The red-dashed line corresponds to an adjusted p-value of 0.01. Species or strains significantly increased in control subjects plot in the upper right quadrant, whereas those decreased in control subjects plot in the upper left quadrant. Panels (a,b) correspond to species and strain-level findings, respectively. (c) The average number of cohorts in which a significant DA strain or species is detected and plotted. Green and pink represent the number of cohorts supporting strains that are significantly increased and decreased in controls as compared to case subjects, respectively. [file Image_6.TIFF]

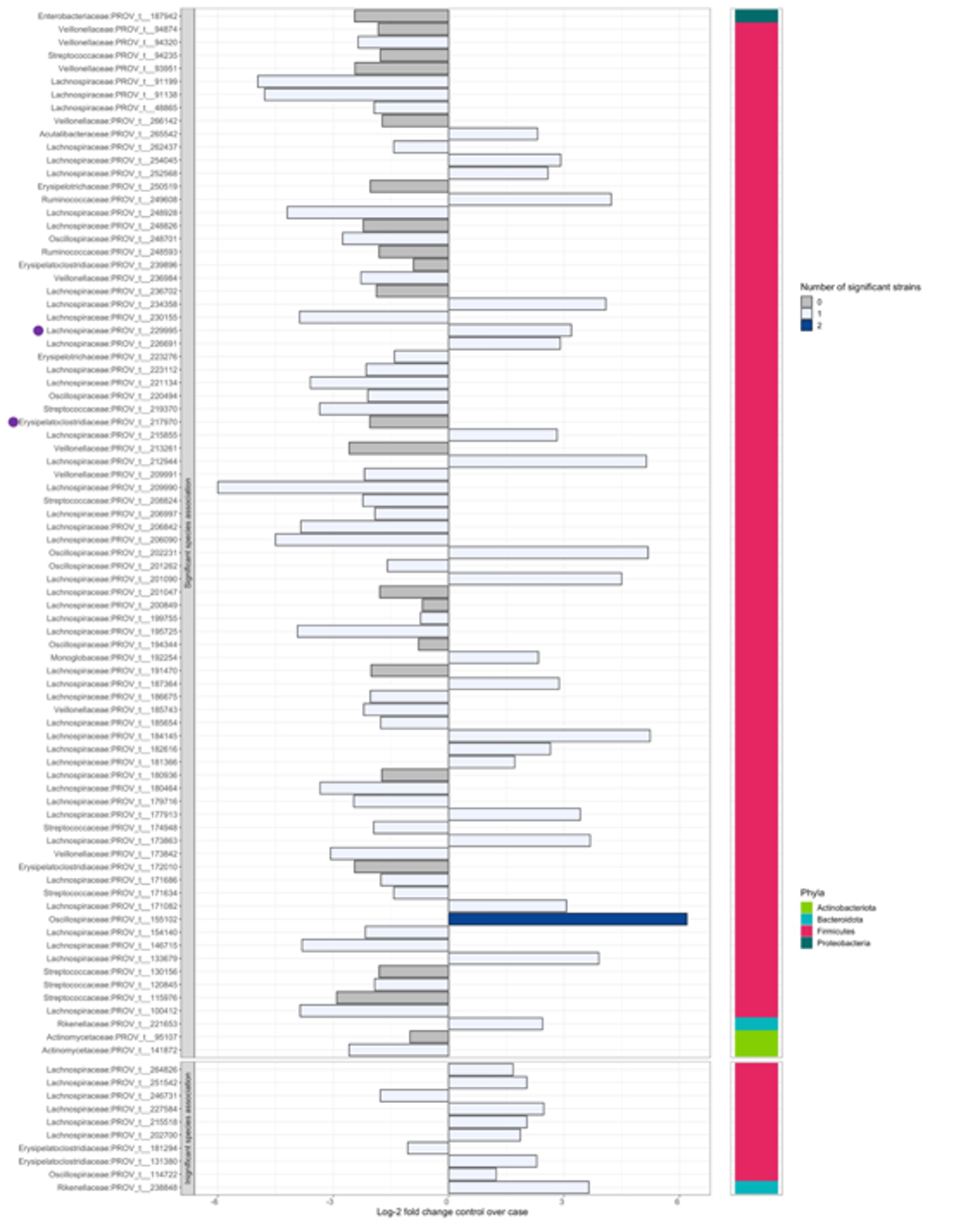

Supplement: Supplementary Figure 7 — Multi-technology meta-analysis identifies species and strains that are yet to be named even at the species-level that are differentially abundant in stool samples from Crohn’s as compared to control subjects. Left panel plots the log-2 fold change of differential abundance aggregated at the species level. Bars are colored by the number of strains within a species that are significantly DA with gray bars indicating cases where only species-level significance is observed. Right strip indicates phylum-level placement of each species. Top panel plots cases with species-level significant differences with or without strain-level significant differences. Bottom panel plots cases with only strain-level but no species-level significant differences. Significance was determined at adjusted p < 0.01. Purple dots point to species decreased in both stool and mucosa of controls compared to CD patients. Only species that are not taxonomically named are shown. [file Image_7.TIFF]

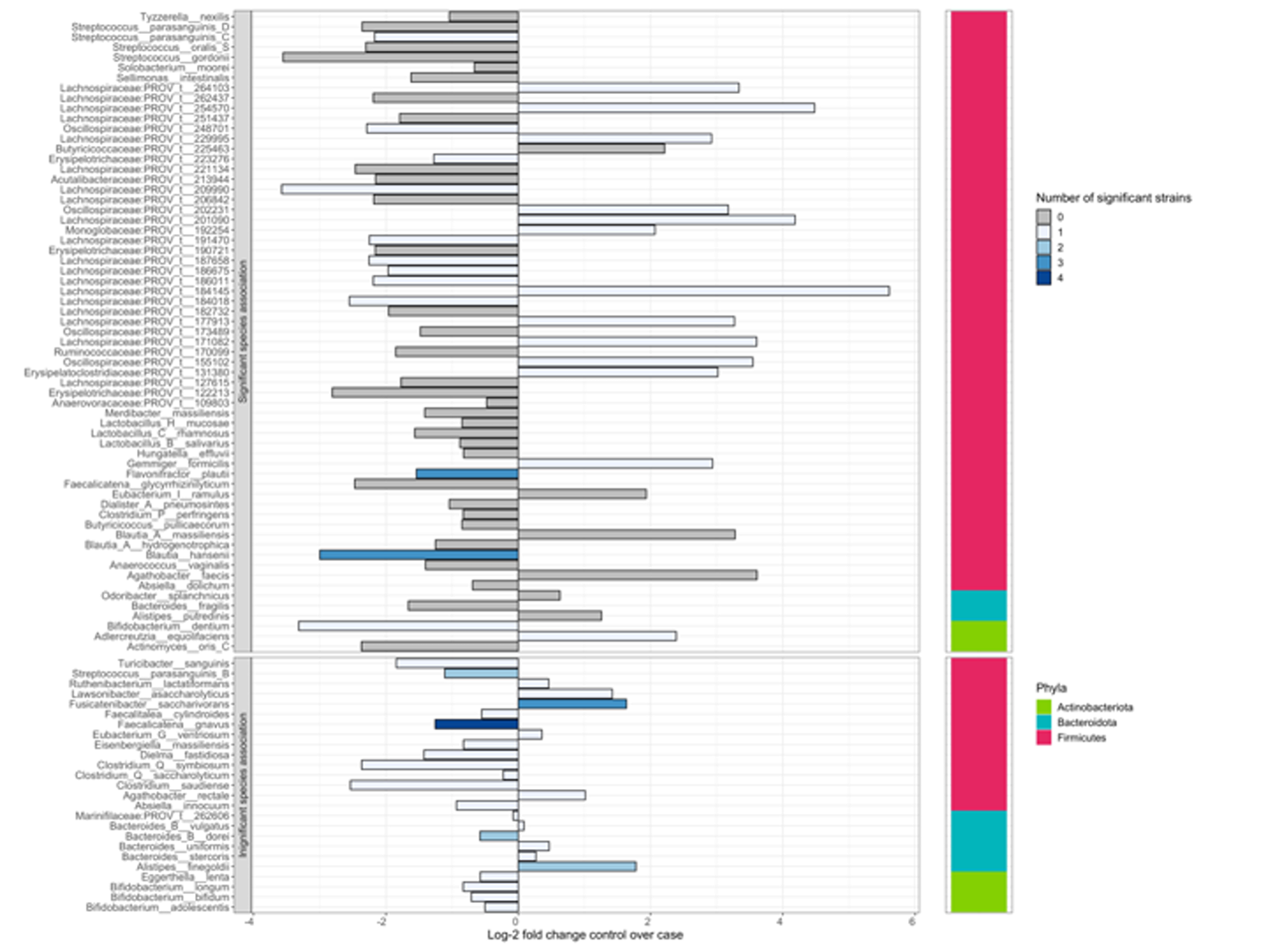

Supplement: Supplementary Figure 8 — Novel dysbiosis and homeostasis-associated species and strains in the gut lumen of Ulcerative colitis subjects that were previously unreported in isolated analyses. Left panel plots the log-2 fold change of differential abundance aggregated at the species level. Bars are colored by the number of strains within a species that are significantly differentially abundant with gray bars indicating cases where only species-level significance is observed. Right strip indicates phylum-level placement of each species. Top panel plots cases with species-level significant differences with or without strain-level significant differences. Bottom panel plots cases with only strain-level but no species-level significant differences. Significance was determined at adjusted p < 0.01. [file Image_8.TIFF]

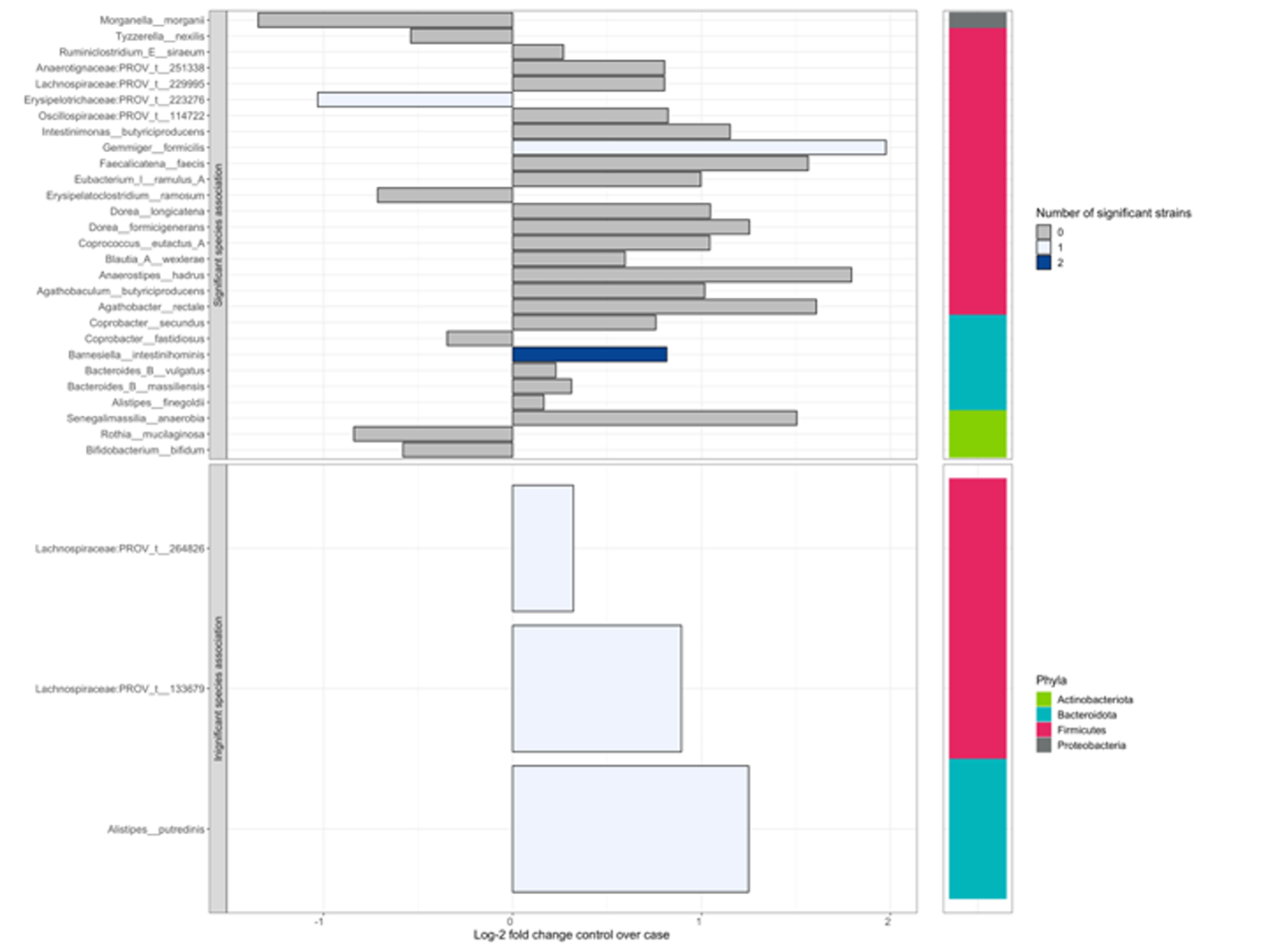

Supplement: Supplementary Figure 9 — Novel dysbiosis and homeostasis-associated species and strains in the mucosa of Crohn’s disease subjects that were previously unreported in isolated analyses. Left panel plots the log-2 fold change of differential abundance aggregated at the species level. Bars are colored by the number of strains within a species that are significantly differentially abundant with gray bars indicating cases where only species-level significance is observed. Right strip indicates phylum-level placement of each species. Top panel plots cases with species-level significant differences with or without strain-level significant differences. Bottom panel plots cases with only strain-level but no species-level significant differences. Significance was determined at adjusted p < 0.01. [file Image_9.TIFF]

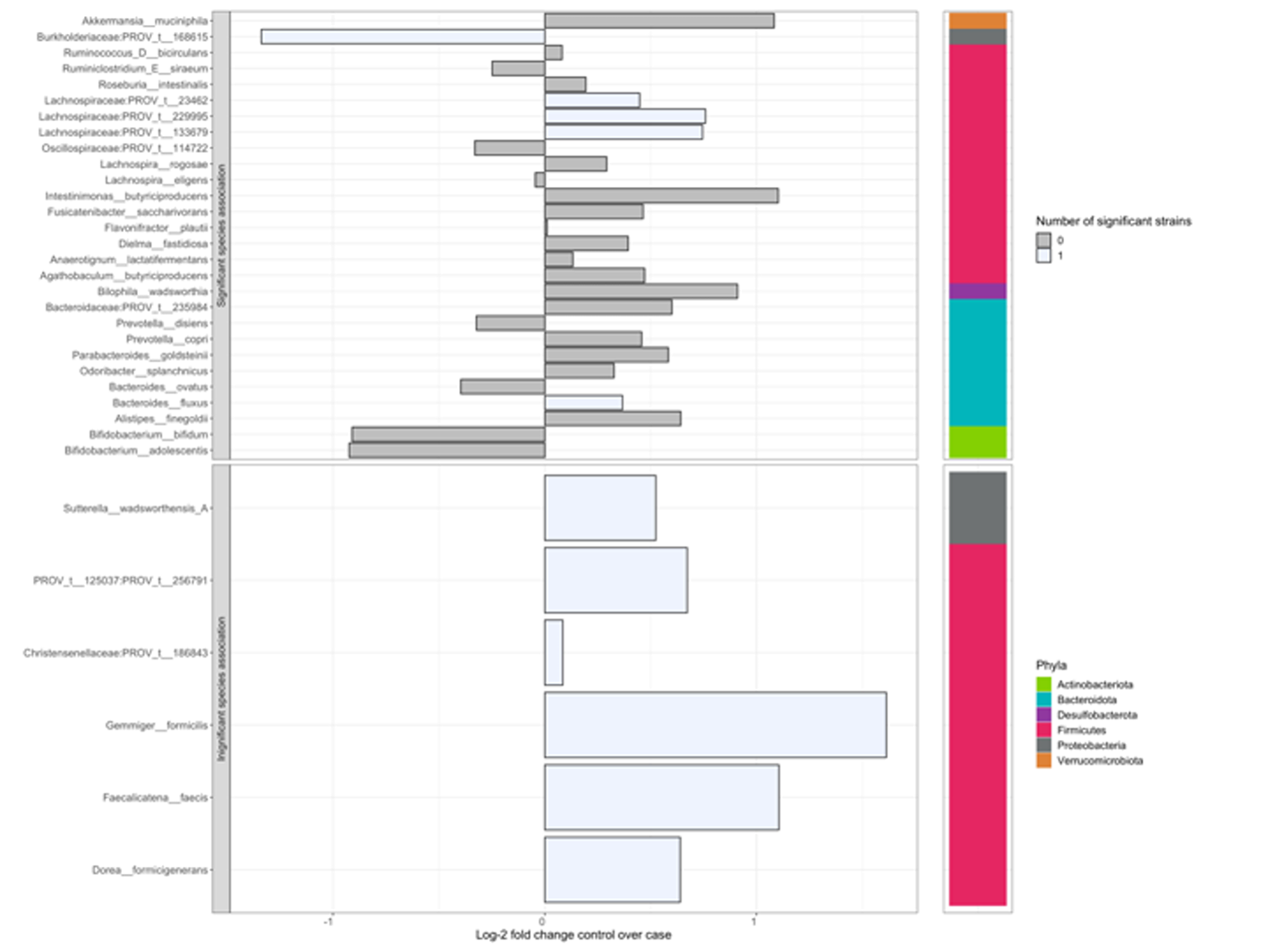

Supplement: Supplementary Figure 10 — Novel dysbiosis and homeostasis-associated species and strains in the mucosa of Ulcerative Colitis subjects that were previously unreported in isolated analyses. Left panel plots the log-2 fold change of differential abundance aggregated at the species level. Bars are colored by the number of strains within a species that are significantly differentially abundant with gray bars indicating cases where only species-level significance is observed. Right strip indicates phylum-level placement of each species. Top panel plots cases with species-level significant differences with or without strain-level significant differences. Bottom panel plots cases with only strain-level but no species-level significant differences. Significance was determined at adjusted p < 0.01. [file Image_10.TIFF]

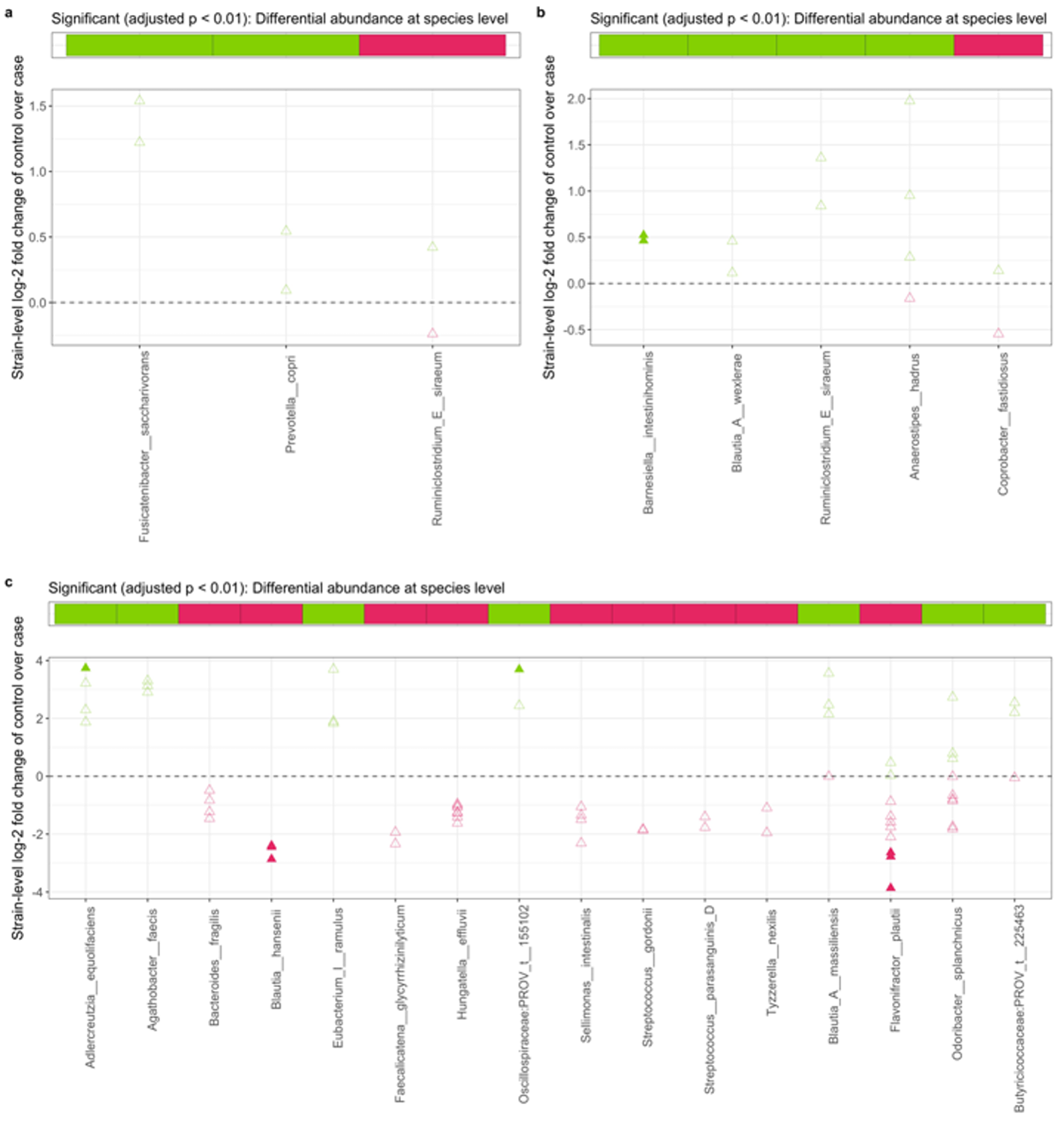

Supplement: Supplementary Figure 11 — Strain-identity even within a species is important to the functional role of a bacterium in IBD. Top strip plots enrichment (green) or decrease (pink) of species in controls as compared to case subjects. Bottom panel plots the log-2 fold change of strains within a species with closed and open triangles indicating significant and non-significant findings in strain-level MTMA, respectively. Significance was determined at an adjusted p < 0.01. Panels (a) through (c) summarize findings for controls compared to UC in mucosa, controls compared to CD in mucosa, controls compared to UC in stool, respectively. [file Image_11.TIFF]

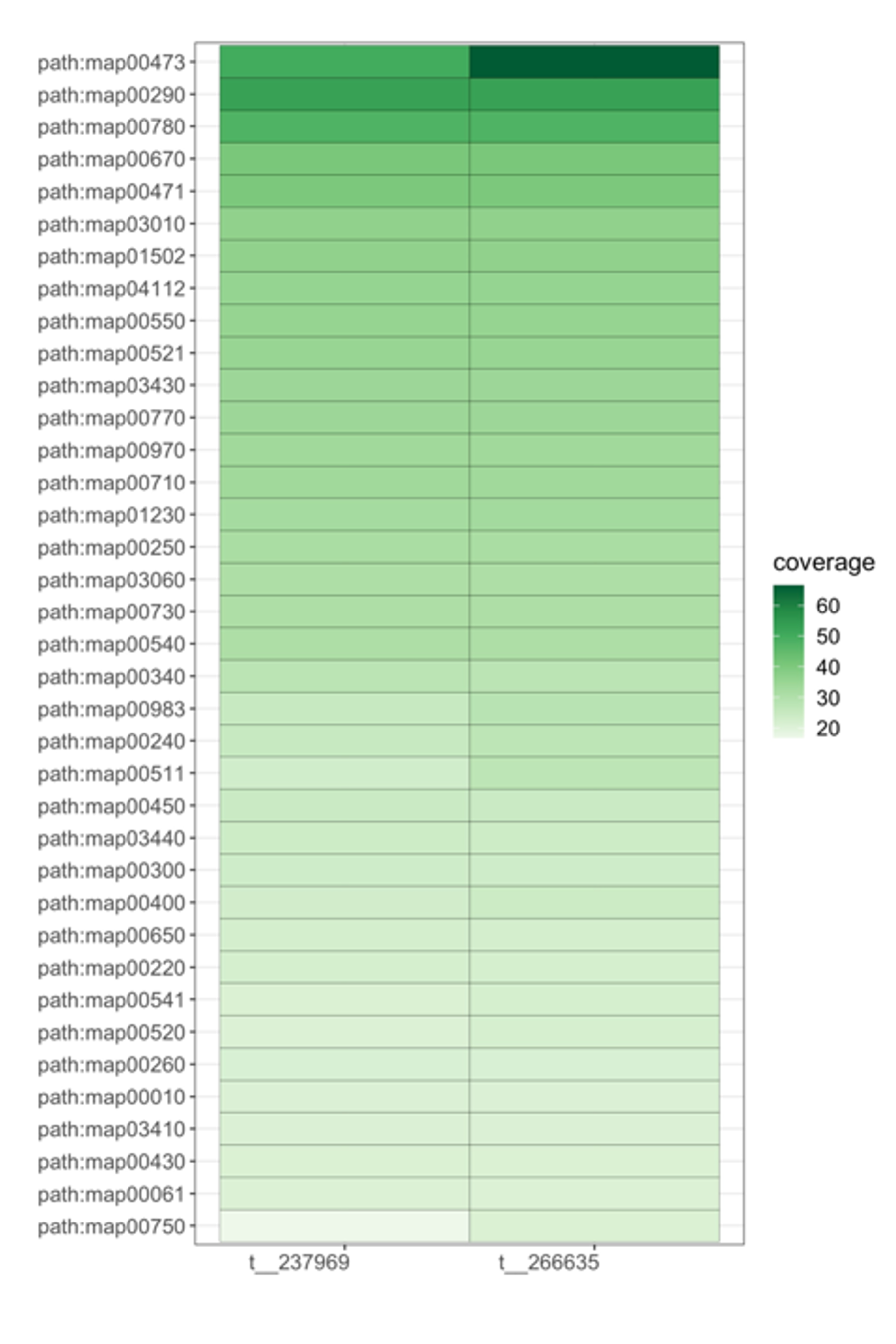

Supplement: Supplementary Figure 12 — Strains within a species demonstrate differences in their functional potential. KEGG-pathway profiles of two Odoribacter splanchnicus strains that were identified as significantly enriched (t__237969) and decreased (t__266395) in stool from CD as compared to control subjects by MTMA are shown. Color gradient in the heat map represents the percent coverage of known genes in the pathway that are identified in the genome of the strain. All pathways supported by the presence of at least 20% of the known genes constituting the pathway in one of the strains are shown. [file Image_12.TIFF]

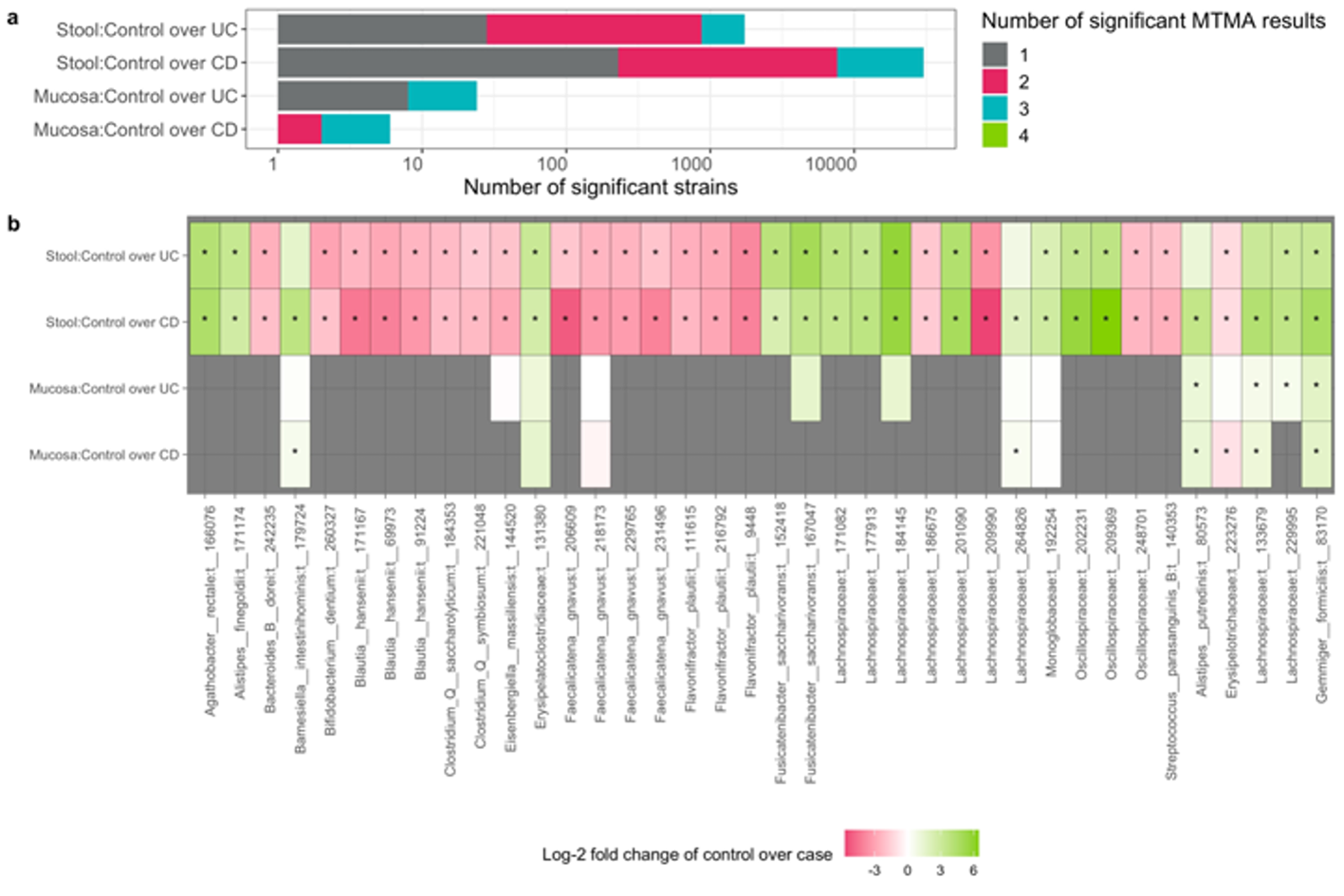

Supplement: Supplementary Figure 13 — Multi-technology meta-analysis identifies strain-associations that are often specific to a disease subtype and microbial ecosystem with few strains that demonstrate significant associations in multiple MTMAs. (a) Number of strains that are significantly DA by MTMA in each of the two subtypes (Controls compared to UC or CD) for each gut-microbial ecosystem (stool/luminal and mucosa) with sub-tallies colored according to the number of MTMAs a strain is significant. (b) MTMA-derived log-2 fold changes for strains identified as significantly (denoted by asterisks) DA in two or more contrasts by MTMA are shown. Significance was determined at adjusted p < 0.01. [file Image_13.TIFF]

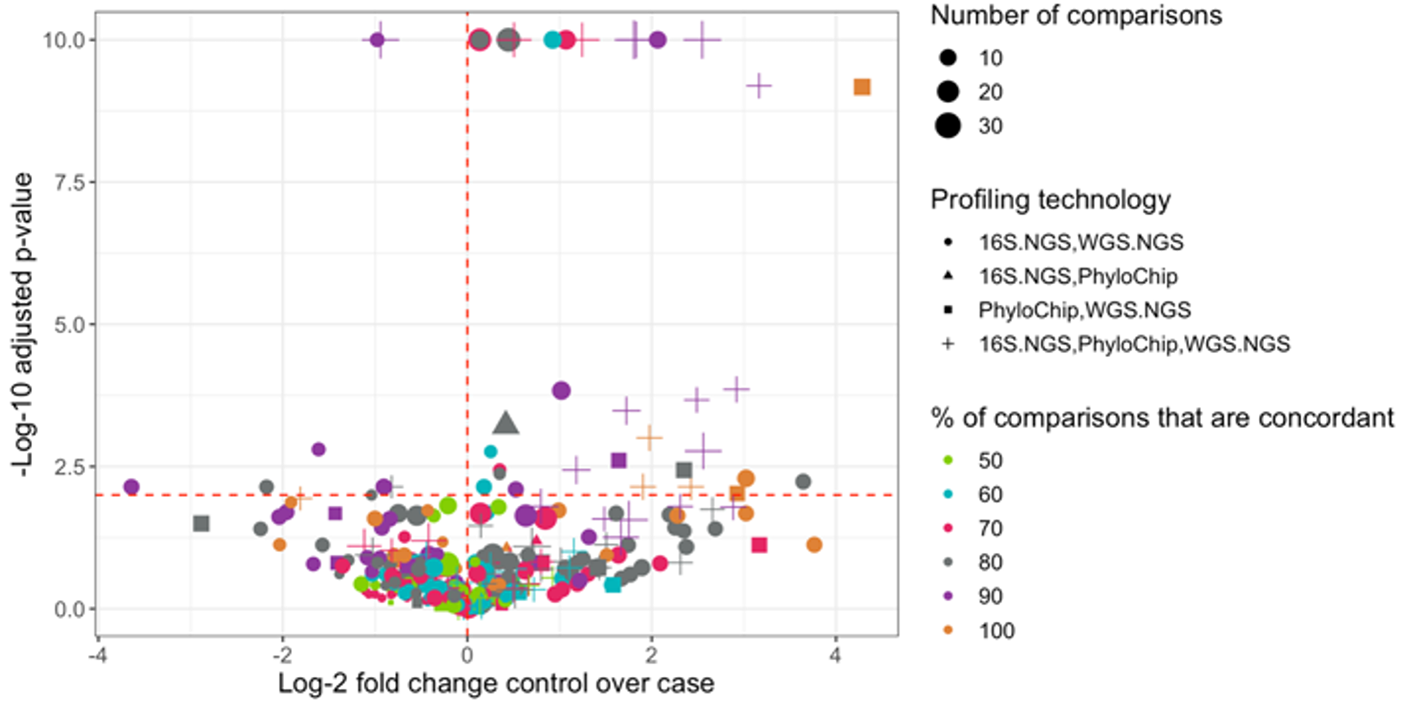

Supplement: Supplementary Figure 14 — Multi-technology meta-analysis identifies greater number of homeostasis as compared to dysbiosis-associated strains in IBD that are consistently decreased across disease subtypes, gut-microbial ecosystems, and DNA-profiling technologies. MTMA-derived adjusted p-values and log-2 fold changes are plotted from an MTMA integrating all IBD datasets described here in. Data points are colored according to the proportion of comparisons in which a strain demonstrated concordance in direction of log-2 fold change and shaped by the DNA-profiling technologies used for characterization of the datasets. The red-dashed line corresponds to an adjusted p-value of 0.01. Strains significantly enriched in controls compared to IBD subjects plot in the upper right quadrant, whereas those decreased in control subjects plot in the upper left quadrant. [file Image_14.TIFF]

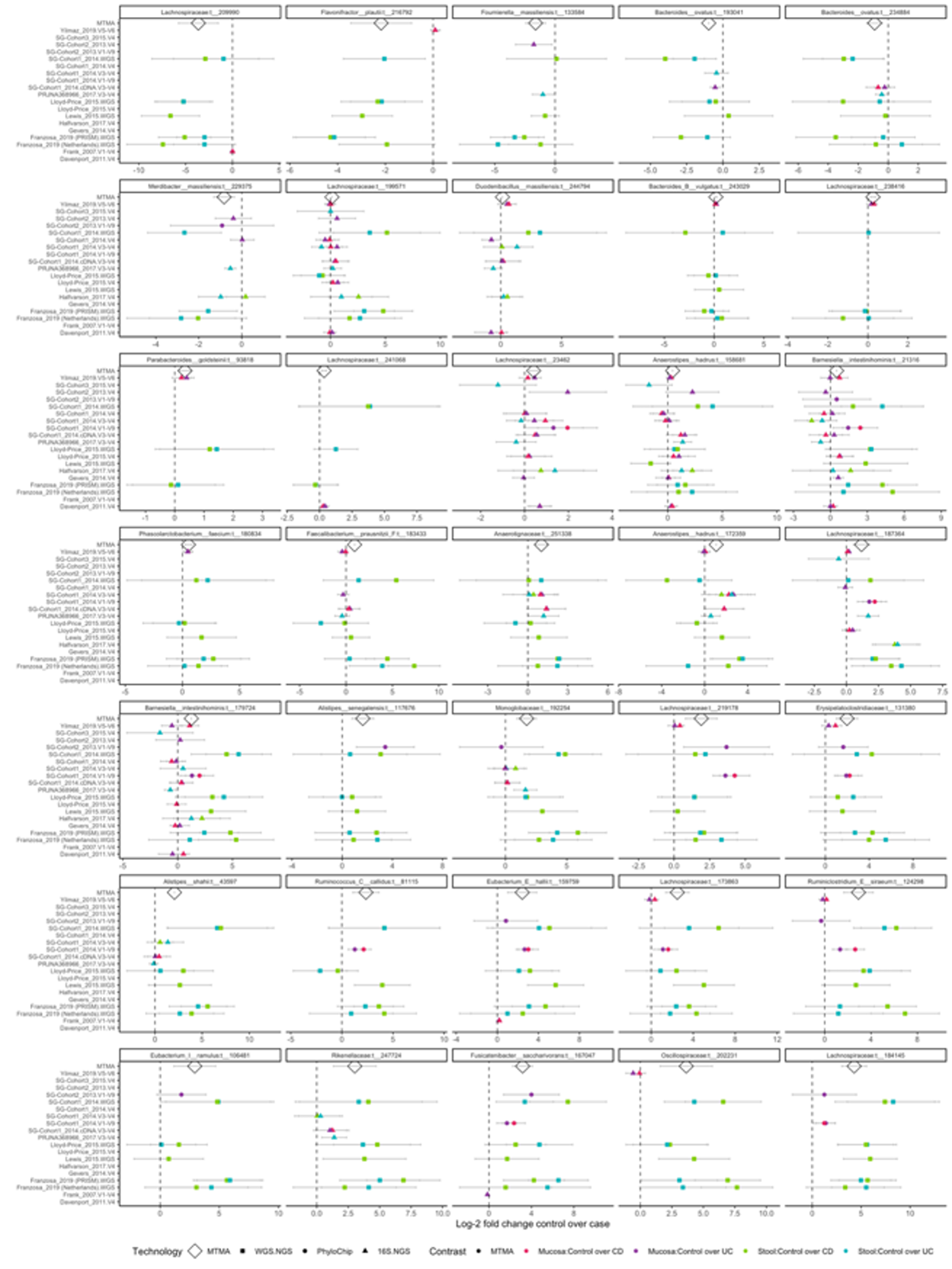

Supplement: Supplementary Figure 15 — Multi-technology meta-analysis identifies strains that are consistently associated (enriched or decreased) with IBD across disease subtypes, gut-microbial ecosystems, cohorts and DNA-profiling technologies. Each panel plots log-2 fold change from MTMA and isolated datasets for strains that are significantly DA in an MTMA integrating all datasets and comparisons described here in. For isolated datasets, points are shaped by the DNA-profiling technology used to characterize the microbiome and colored by gut-microbial ecosystems and disease subtypes being compared to controls. Error bars correspond to the 95% confidence interval. Significance was determined at an adjusted p < 0.01. Plots summarizing results for strains that are detected in less than 75% of the contrasts integrated into the MTMA are shown here. [file Image_15.TIFF]
