# Supplementary material for: Strain level and comprehensive microbiome analysis in inflammatory bowel disease via multi-technology meta-analysis identifies key bacterial influencers of disease
Source: Front Microbiol. 2022 Oct 14;13:961020. doi: 10.3389/fmicb.2022.961020 (PMC9614153; doi:10.3389/fmicb.2022.961020)
Supplement: Supplementary Table 1 — Details pertaining to cohorts, datasets, and comparisons analyzed. [file Table_1.docx]

**Supplementary** **Table 1:** Details pertaining to cohorts, datasets, and comparisons analyzed.

| **Cohort** | **Bio-specimen type** | **Counts (Control-UC-CD)** | **Target region** | **DNA-profiling technology** | **Number of reads Median (range)** |
| --- | --- | --- | --- | --- | --- |
| Gevers_2014 (Gevers et al., 2014) | Mucosa | 109-30-165 | V4 | Illumina MiSeq | 14,359.5 (249 – 100,657) |
| Franzosa_2019 (Franzosa et al., 2019) | Stool | 34-53-68 | WGS | Illumina HiSeq 2500 | PRISM cohort: 16,222,955 (621,560 – 70,940,336) |
|  |  | 22-23-20 |  |  | Netherlands cohort: 11,432,383 (2,044,104 – 29,863,893) |
| PRJNA368966_2017 | Stool | 32-33-0 | V3-V4 | Illumina MiSeq | 37,508 (1,488 – 84,463) |
| Morgan_2012 (Morgan et al., 2012) | Stool | 19-48-63 | V3-V5 | 454 Genome Sequencer FLX Titanium | 1,124.5 (12 – 2,563) |
|  | Mucosa | 9-27-34 |  |  | 744 (39 – 5,652) |
| Lloyd-Price_2015 dbGaP phs001626.v1.p1 | Stool | 36-48-92 | WGS | Illumina HiSeq 2000 | 16,800,769.5 (651,795 – 29,128,040) |
|  | Mucosa | 22-22-36 | V4 | Illumina MiSeq | 27,487 (924 – 52,466) |
| Davenport_2011 (Davenport et al., 2014) | Mucosa | 27-14-13 | V4 | Illumina MiSeq | 7,764.5 (234 – 14,827) |
| Halfvarson_2017 (Halfvarson et al., 2017) | Stool | 9-60-49 | V4 | Illumina HiSeq 2000 | 318,414 (4,462 – 2,935,368) |
| SG-Cohort1_2014 (Laserna-Mendieta et al., 2018; Ryan et al., 2020) | Stool | 7-29-18 | WGS | Illumina HiSeq | 13,621,400 (4,529,515 – 22,256,346) |
|  | Stool | 7-29-18 | V3-V4 | Illumina MiSeq | 40,062 (1,081 – 73,627) |
|  | Mucosa | 15-93-55 | V4 | Illumina MiSeq | 46,384 (2,560 – 559,465) |
|  | Mucosa | 31-91-54 | V3-V4 | Illumina MiSeq | 9,090 (879 – 45,666) |
|  | Mucosa | 25-91-49 | V3-V4 (cDNA) | Illumina MiSeq | 23,511 (9,942 – 84,489) |
|  | Mucosa | 25-86-54 | V1-V9 | G4 PhyloChip | *Does not apply* |
| SG-Cohort2_2013 *generated in this study* | Mucosa | 20-20-0 | V4 | Illumina MiSeq | 328,474.5 (18,819 – 639,650) |
|  |  | 20-20-0 | V1-V9 | G3 PhyloChip | *Does not apply* |
| SG-Cohort3_2015 (Yin et al., 2020) | Stool | 15-10-0 | V4 | Illumina MiSeq | 82,530 (58,358 – 142,226) |
| Zhou_2018 (Zhou et al., 2018) | Stool | 95-58-104 | V4 | Illumina MiSeq | 4,806 (291 – 11,021) |
| Yilmaz_2019 (Yilmaz et al., 2011) | Mucosa | 158-147-189 | V5-V6 | Ion Torrent PGM | 18,751.5 (3,230 – 1,469,000) |
| Frank_2007 (Frank et al., 2007) | Mucosa | 36-33-21 | V1-V4 | Amersham MegaBACE 1000 | 60 (23 – 94) |
| Lewis_2015 (Lewis et al., 2015) | Stool | 26-0-105 | WGS | Illumina HiSeq 2000 | 13,852,376.5 (771,813 – 43,255,840) |
